# Synthesis, antidiabetic evaluation, and computational modeling of 3-acetyl-8-ethoxy coumarin derived hydrazones and thiosemicarbazones

**DOI:** 10.1039/d5ra04619j

**Published:** 2025-10-16

**Authors:** Wajeeha Zareen, Nadeem Ahmed, Ali Muhammad Khan, Suraj N. Mali, Nastaran Sadeghian, Naflaa A. Aldawsari, Parham Taslimi, Abdullah K. Alanazi, Muhammad Tahir, Mussarat Tasleem, Zahid Shafiq

**Affiliations:** a Institute of Chemical Sciences, Bahauddin Zakariya University 60800 Multan Pakistan zahidshafiq@bzu.edu.pk; b College of Chemistry & Chemical Engineering, Central South University Changsha Hunan 410083 China; c School of Pharmacy, D.Y. Patil University (Deemed to be University) Sector 7, Nerul Navi Mumbai 400706 India; d Department of Biotechnology, Faculty of Science, Bartin University 74110 Bartin Turkiye; e Chemistry Department, Al-Khurmah University College, Taif University Al-Khurmah 21985 Saudi Arabia; f Department of Chemistry, College of Science, Taif University Taif Saudi Arabia

## Abstract

Inhibiting important enzymes like α-amylase and α-glucosidase is essential for controlling hypoglycemia and its related complications in diabetes mellitus. A series of novel hydrazones and thiosemicarbazones have been synthesized and evaluated for their ability to inhibit enzymes, causing hypoglycemia and diabetes mellitus in the human body. From synthesized compounds, compound 3b from the carbohydrazide series, demonstrated the strongest potency against α-amylase and α-glucosidase, with respective IC_50_ values of 252.45 ± 12.81 nM and 159.10 ± 8.15 nM and in the case of the carbothioamide series, thiosemicarbazone 5e, exhibited the highest inhibitory potency, with IC_50_ values of 73.68 ± 2.84 nM for α-glucosidase and 146.18 ± 7.35 nM for α-amylase. These compounds were compared to the standard drug acarbose with IC_50_ values of 315.74 ± 15.06 nM and 437.93 ± 13.96 nM for α-glucosidase and α-amylase. Novel compounds having a variety of structural configurations, showed encouraging activity profiles with potent inhibition of α-amylase and α-glucosidase. The interactions between these inhibitors and the target enzyme's active sites were further examined by doing Density Function Theory (DFT), molecular docking, and structure–activity relationship (SAR) studies, which provides information about the derivatives that are more potent. Toxicity, metabolism, and drug-likeness characteristics of newly synthesized hydrazones and thiosemicarbazones were investigated by *in silico* ADMET tests.

## Abstract 2

Inhibiting important enzymes like α-amylase and α-glucosidase is essential for controlling hypoglycemia and its related complications in diabetes mellitus.

## Graphical abstract

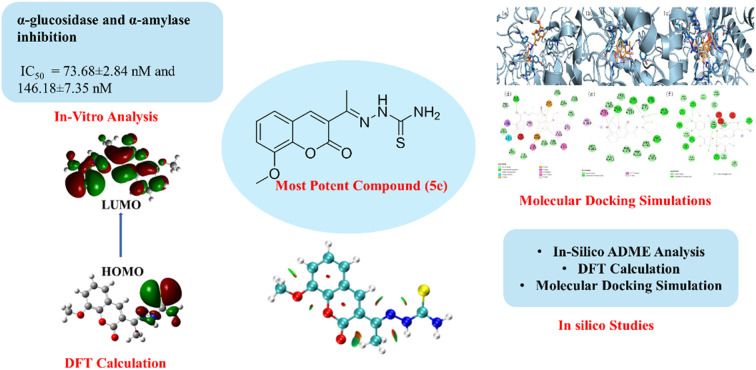

## 1. Introduction

Diabetes mellitus (DM) has prevailed as a serious global health issue and a chronic illness due to its numerous repercussions and high prevalence. Diabetes mellitus (DM) is affecting more than 400 million people around the world and is expected to reach 693 million by 2045.^1,2^ It's a metabolic disorder in which a person has high blood sugar for an extended period of time due to inadequate insulin secretion or action.^3^ There are different categories of diabetes: Type 1, Type 2, gestational diabetes, and other specific forms such as cystic fibrosis-related and drug-induced diabetes.^4^ Hyperglycemia decreases the rate of glucose transport across tissues due to raised levels of α-glucosidase, an enzyme responsible for hydrolysis of carbohydrates into monosaccharides.^5^ The use of inhibitors of carbohydrate-digesting enzymes such as α-glucosidase and α-amylase is a significant therapeutic strategy for the control of postprandial blood glucose level. Acarbose, voglibose, and miglitol are commonly used inhibitors, but they often cause side effects, such as flatulence, abdominal pain, and diarrhea, which limits their use in patients having gastrointestinal or hepatic diseases.^6^ Diabetic complications, like increased oxidative stress, alteration in the antioxidant defense system, and dyslipidemia lead to serious issues such as renal damage, neuropathy, cerebrovascular disease, and limb amputation, which make diabetes more fatal.^7^ Regarding treatment methods, insulin therapy and oral hypoglycemic agents remain the primary modes of intervention, which consider the control of blood glucose levels, while changes to dietary options are in testing, including alternative medicines like plant-based syrups. Recent studies report the use of Chinese medicines in type 2 diabetes mellitus (T2DM) *via* intestinal microbiota modulation to support future clinical applications. Astragalus improves intestinal barrier function and immunity by acting on intestinal microbiota to treat T2DM.^8,9^ The limitations of existing treatments illustrate the need to devise new treatment strategies that are more effective and have fewer undesirable consequences, creating a need to focus on deepened research in the management of diabetes care.

Among the naturally occurring bioactive compounds, coumarins have drawn great attention to themselves due to the wide range of their pharmaceutical applications and their harmless nature, serving as a valuable pharmacophore to be used in a drug design. Coumarins are also known as 2*H*-chromen-2-ones, and they make up a large group of heterocyclic compounds that contain a fused ring structure of benzene and 2-pyrone, making this group structurally unique.^10^ Coumarins exhibit a range of pharmacological activities including anti-diabetic,^11–13^ antioxidant,^14^ antibacterial,^15^ antifungal,^16^ antiviral, anti-inflammatory, and anticancer properties,^17^ highlighting their biological significance. Furthermore, coumarin derivatives exhibit significant inhibition of α-glucosidase and α-amylase, suggesting their potential utility in diabetes management through the modulation of postprandial glucose levels.^18,19^ Numerous studies have demonstrated that coumarin-based compounds inhibit carbohydrate-digesting enzymes, thus reducing hyperglycemia and serving as potential candidates for anti-diabetic drug development. The Coumarin–Quinazolinone (CQ) scaffold is reported to target specific cellular organelles, resulting in significant enhancement in the efficacy of photodynamic therapy.^20^

Furthermore, hydrazones, a subset of azomethine compounds, are defined by their distinct C=N linkage and are categorized under Schiff bases having an alkyl or aryl group directly attached to the azomethine nitrogen.^7,21^ The scaffold has been deeply investigated for its pharmaceutical properties, as they possess anti-inflammatory,^22^ anticancer,^23^ anti-tyrosinase,^24^ anticonvulsant,^25^ antioxidant, antifungal and antibacterial activities.^22^ Additionally, hydrazone derivatives have shown potential in the treatment of diabetes, particularly due to their inhibition of α-glucosidase and α-amylase. Hydrazones can potentially improve glycemia regulation by changing the metabolism of carbohydrates, thereby decreasing post-meal rises in the blood glucose levels. Their structural flexibility allows for extensive functionalization, making it possible to design highly selective and effective enzyme inhibitors. The addition of hydrazone moieties into therapeutic scaffolds has been studied to enhance their enzyme inhibition, their bioavailability and their pharmacokinetic properties.^26–28^ Because of their multi-target nature and strong enzyme binding ability, hydrazones offer a unique class of compounds to focus on for further research in anti-diabetic drug development. They offer a practical approach for the formation of more efficient and less toxic inhibitors of α-glucosidase and α-amylase useful in the treatment of diabetes.

**Fig. 1:**
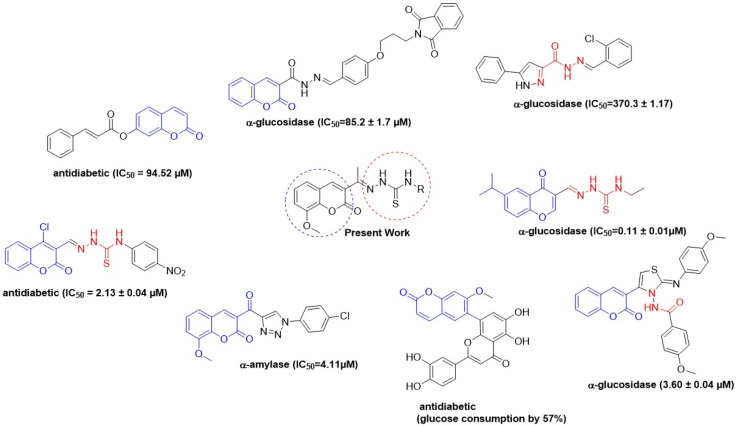
Structure of the previously reported chromone, hydrazone and thiosemicarbazone derivatives as antidiabetic agents.

Moreover, thiosemicarbazones, with the general formula R_1_R_2_C=N–NH(C=S)NHR, have recently gained attention in medicinal chemistry because of a wide range of biological activities such as antibacterial,^29^ antifungal,^30^ antiviral,^31^ anticancer,^32^ antihelminthic, and antimalarial activities.^33^ Out of all biological activities, these compounds have shown significant anti-diabetic activity along with numerous other pharmacological activities. The –C=N–NH–SH functional group has peculiar electronic characteristics due to the sulfur atom which is responsible for enhancing the enzyme inhibitory activity these compounds display. Recent research shows that thiosemicarbazone derivatives are potent enzyme inhibitors of α-glucosidase and α-amylase, the major enzymes responsible for the digestion of carbohydrates.^4,34^ These inhibitors reduce the breakdown of complex carbohydrates into simple sugars, thus reducing glucose levels in the blood after eating. Managing postprandial glucose levels is crucial in effective diabetes treatment. Recently developed thiosemicarbazone derivatives as α-glucosidase and α-amylase inhibitors are a breakthrough in diabetes control, as illustrated in Fig. 1.

Treatment of diabetes with traditional medicines is known to cause many unwanted effects, thus proving a need for other compounds with better results and lower side effects. Conventional treatments often have side effects, which emphasize the importance of looking at alternative molecules with better efficacy and less toxicity. Promising options are thiosemicarbazones, hydrazones, and coumarins, which have a great ability to control carbohydrate digestion and lower postprandial glucose surges. Pharmacophore hybridization is an efficient drug design strategy that combines multiple pharmacophoric features into a single molecule to improve binding affinity and therapeutic efficacy.^35^ Aiming to improve the knowledge of inhibitory processes and structural design, *in silico* studies have been performed to assess binding interactions with important enzymes. Molecular docking simulations were performed to comprehend the binding affinities and the interaction mechanisms of these drugs with the active sites of α-glucosidase and α-amylase, therefore offering understanding of their selectivity and potency. By means of *in silico* analyses combined with experimental data, rational design of next-generation anti-diabetic drugs is facilitated, therefore offering a complete framework for the evolution of more efficient and safer treatments for diabetes.

## 2. Experimental

### 2.1. Material and methods

All chemicals including, 4-methyl phenyl isothiocyanate, 4-benzyl phenyl isothiocyanate, 2,4 methyl phenyl isothiocyanate, 1-isothiocyanato-2-methylpropane, thiosemicarbazide, thiophene-2-carboxylic acid, 4-fluorobenzoic acid, 4-bromobenzoic acid, 2-methoxy benzoic acid, pyrazine-2-carboxylic acid, 1*H*-indole-3-carboxylic acid, furan-2-carboxylic acid, 3-methoxy salicylaldehyde, 3-ethoxy salicylaldehyde, hydrazine mono hydrate, MeOH, ethyl acetate, CH_3_COOH used in the current study was purchased from Sigma-Aldrich (Germany) and was further refined. NMP spectra were obtained in DMSO-d_6_ using Bruker Advance 400 spectrometers. The stability of synthesized compound in DMSO is checked through UV-vis (SI). Chromatographs were examined under UV light irradiation.

### 2.2. Molecular docking analysis

Considering the background literature^36–40^ on thiosemicarbazone derivatives evaluated for *in vitro* α-glucosidase, and α-amylase inhibitory activities, we thought worthwhile to explore the binding site characteristics for our synthesized set of compounds using molecular simulations. Thus, we carried out molecular docking analysis using ‘AutoDock Vina v1.2.x’.^41^ For target crystal structures, we used α-glucosidase (PDB: 3A4A)^36–40^ and α-amylase (PDB: 3BAJ) protein database IDs. For molecular docking simulations, we followed earlier reported protocol^39^ for optimization and docking runs. Finally, the visualization of docking interactions was done using ‘PyMol’^42^ (Free for academics, https://www.pymol.org/) and ‘Discovery Studio Visualizer’, 2023 (https://discover.3ds.com/discovery-studio-visualizer-download).

### 2.3. ADME analysis

We evaluated the compound's drug similarity, lipophilicity, medicinal chemistry,^43,44^ and pharmacokinetics using the ‘SwissADME’^45^ website (http://www.swissadme.ch/). This tool provides comprehensive and reliable predictions of the compounds' ADMET properties, aiding in the identification and development of new drugs.

### 2.4. DFT study

DFT is often used to investigate the electrical characteristics of potential synthetic drugs.^46^ The Becke, Lee–Yang–Parr (B3LYP) exchange-correlation functional, and 6-311+G(d,p) basis set were incorporated into the Gaussian 09 software to optimize the geometry of all the chemicals under investigation in the gaseous phase. To forecast the chemical reactivity of each of these compounds, the energy gap (Δ*E*) of FMO-Frontier Molecular Orbitals—which include the HOMO (highest occupied molecular orbital) and the LUMO (lowest unoccupied molecular orbital)—was calculated. Koopman's theorem^47^ was used to derive the other significant electronic properties, such as the electrophilicity index (*ω*), chemical potential (*μ*), softness (*S*), and hardness (*η*). Using GaussView, the geometry of every compound under investigation was displayed.

### 2.5. Antidiabetic assays

#### 2.5.1 α-Glycosidase studies

The compounds' α-glycosidase inhibitory action was created using Tao *et al.*'s^48^ methodology. The substrate was *p*-nitrophenyl-d-glucopyranoside (*p*-NPG). Initially, 20 μL of α-glycosidase solution (0.15 eU per mL) produced in phosphate buffer (pH 7.4, 5 mM) and 5 μL of various chemical concentrations were mixed with 75 μL of phosphate buffer solution (5 mM, pH 7.4). After 10 minutes of preincubation at 35 °C, *p*-NPG was added to start the reaction. Additionally, following a brief incubation time at 35 °C, 20 μL of *p*-NPG was transferred in phosphate buffer (pH 7.4, 5 mM). At 405 nm, the absorbances were measured. The amount of enzyme that catalyzes the hydrolysis of 1.0 mol *p*-NPG per minute at pH 7.4 is one α-glycosidase unit.^49,50^

#### 2.5.2 α-Amylase studies

Using starch as a substrate, the compounds' α-Amylase inhibitory activities were achieved in accordance with Xiao's^51^ methodology. A 0.4 M starch combination was made in 80 mL of NaOH solution and heated to 80 °C for 30 minutes in order to create the starch. After that, 10 μL of various chemical concentrations were combined with 35 μL of starch solution and 35 μL of phosphate buffer (pH 6.9), and the mixture was incubated for 25 minutes at 35 °C. Finally, the final combination was reincubated for 25 minutes after 20 μL of the α-amylase solution was added. Each tube was filled with 50 μL of HCl (0.1 M) to complete the reactions. At 580 nm, the absorbance was measured. The amount of αamylase needed to release one μmol of reducing sugar, which is estimated to be glucose per minute at pH 6.9 and 40 °C, is known as one α-amylase unit.^52,53^

### 2.6. Antioxidant assay (metal chelating)

Fe^2+^ chelating ability of novel compounds was predicted according to Dinis *et al.*^54^ with slight modification. Fe^2+^-binding capacity of novel compounds was spectrophotometrically recorded at 522 nm. This study was conducted based on previous papers.^55,56^

## 3. Results and discussion

### 3.1. Chemistry

**Scheme 1:**
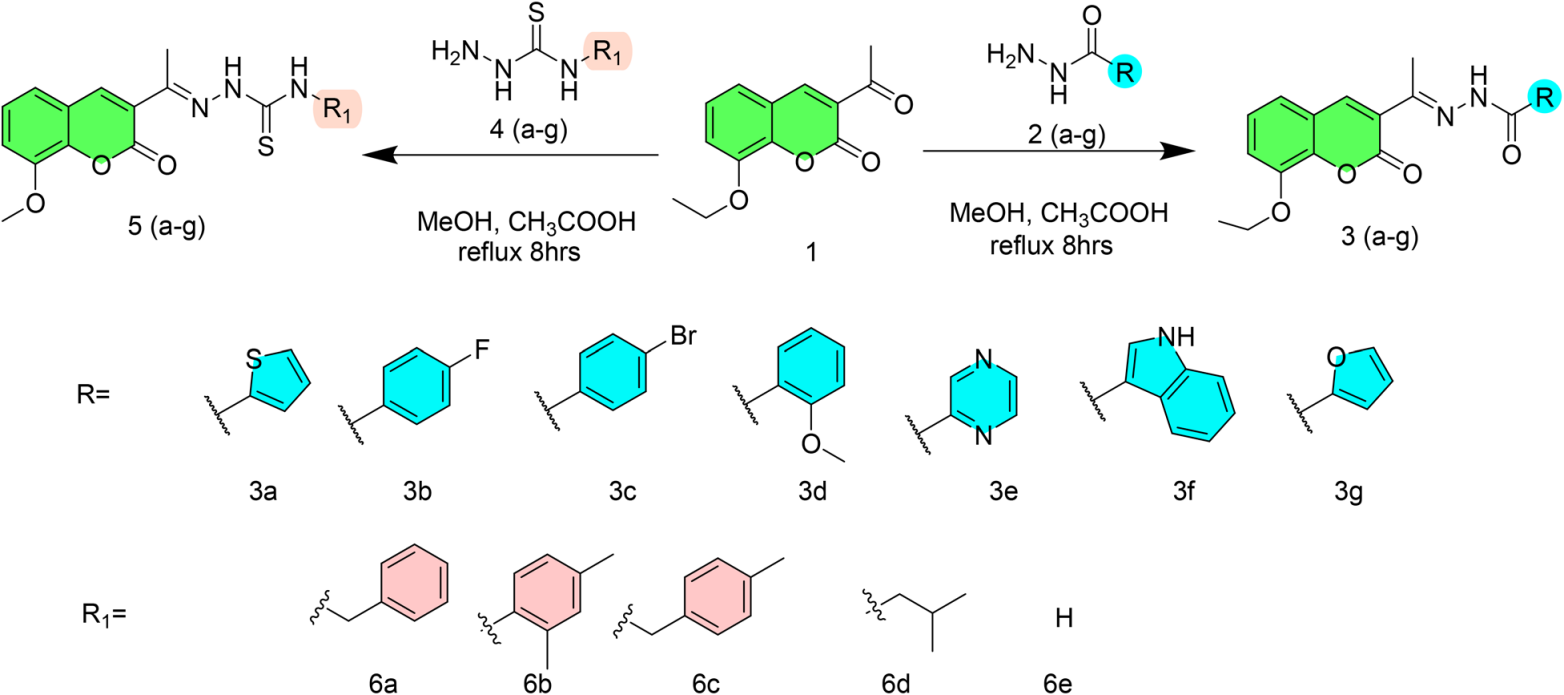
Synthesis of 3(a–g) and 5(a–e).

Coumarin derivatives were synthesized by previously reported method.^57^ Then, coumarin derivatives (1 mmol) were dissolved in anhydrous MeOH (10 mL) by adding 2–3 drops of glacial acetic acid and reaction mixture was set on stirring plate. Appropriate thiosemicarbazide and hydrazide (1 mmol) was added into the reaction mixture and refluxed for 3 h. Reaction progress was monitored by TLC and after completion of reaction, the formed precipitates (3a–g and 5a–e) were collected by filtration, washed with MeOH three times (Scheme 1) and then dried in oven for further studies.

#### 3.1.1 (*E*)-*N*′-[1-(8-Ethoxy-2-oxo-2*H*-chromen-3-yl)ethylidene]thiophene-2-carbohydrazide (3a)

Yield 82%; UV-vis: *λ*_max_ = 426 nm (MeCN); FT-IR: 1577 cm^−1^ (C=N), 1656 cm^−1^ (C=O), 3163 cm^−1^ (NH); ^1^H NMR (400 MHz, DMSO) *δ* 11.04 (1H, s), 8.24 (1H, s), 8.07 (1H, d, *J* = 3.6 Hz), 7.89 (1H, dd, *J* = 5.0, 1.3 Hz), 7.40 (1H, dd, *J* = 7.2, 2.0 Hz), 7.34–7.24 (1H, m), 7.19 (1H, t, *J* = 4.4 Hz), 4.20 (1H, d, *J* = 7.0 Hz), 2.31 (2H, s), 1.42 (1H, s). 13C NMR (101 MHz, DMSO) *δ* 163.71, 159.49, 153.19, 146.00, 142.51, 135.94, 131.71, 130.96, 130.08, 127.61, 127.27, 125.21, 120.80, 119.90, 116.05, 64.92, 17.01, 15.07. HRMS calculated for [M + H], C_18_H_17_N_2_O_4_S^+^: *m*/*z*: 357.0904; found 357.08853.

#### 3.1.2 (*E*)-*N*′-[1-(8-Ethoxy-2-oxo-2*H*-chromen-3-yl)ethylidene]-4-fluorobenzohydrazide (3b)

Yield 85%; UV-vis: *λ*_max_ = 426 nm (MeCN); FT-IR: 1568 cm^−1^ (C=N), 1657 cm^−1^ (C=O), 3270 cm^−1^ (NH); ^1^H NMR (400 MHz, DMSO) *δ* 10.85 (1H, s), 8.19 (1H, s), 7.97 (2H, dd, *J* = 8.7, 5.7 Hz), 7.32 (6H, tdt, *J* = 14.6, 11.0, 7.0 Hz), 4.20 (1H, d, *J* = 7.0 Hz), 2.34 (3H, s), 1.42 (1H, s). 13C NMR (101 MHz, DMSO) *δ* 170.03, 163.72, 159.41, 153.17, 146.78, 143.30, 142.47, 141.49, 135.94, 131.72, 130.96, 130.08, 127.31, 125.22, 120.85, 119.48, 115.21, 56.62, 23.89, 16.34. HRMS calculated for [M + H], C_20_H_18_FN_2_O_4_^+^*m*/*z*: 369.1245; found 369.12245.

#### 3.1.3 (*E*)-4-Bromo-*N*′-[1-(8-ethoxy-2-oxo-2*H*-chromen-3-yl)ethylidene]benzohydrazide (3c)

Yield 80%; UV-vis: *λ*_max_ = 426 nm (MeCN); FT-IR: 1568 cm^−1^ (C=N), 1662 cm^−1^ (C=O), 3268 cm^−1^ (NH); ^1^H NMR (400 MHz, DMSO) *δ* 10.89 (1H, s), 8.16 (1H, d, *J* = 24.4 Hz), 7.84 (1H, d, *J* = 8.1 Hz), 7.73 (1H, d, *J* = 8.1 Hz), 7.41–7.23 (2H, m), 4.20 (1H, d, *J* = 7.0 Hz), 2.34 (2H, s), 1.42 (1H, s). 13C NMR (101 MHz, DMSO) *δ* 177.44, 159.16, 147.54, 146.78, 143.20, 143.06, 140.95, 132.76, 130.21, 126.16, 125.45, 125.19, 124.93, 124.05, 120.80, 119.89, 115.19, 56.63, 40.67, 40.46, 40.25, 40.04, 39.84, 39.63, 39.42, 24.09, 16.68. HRMS calculated for [M + H], C_20_H_18_BrN_2_O_4_^+^*m*/*z*: 429.0444; found 429.04190.

#### 3.1.4 (*E*)-*N*′-[1-(8-Ethoxy-2-oxo-2*H*-chromen-3-yl)ethylidene]-2-methoxybenzohydrazide (3d)

Yield 86%; UV-vis: *λ*_max_ = 426 nm (MeCN); FT-IR: 1562 cm^−1^ (C=N), 1654 cm^−1^ (C=O), 3271 cm^−1^ (NH); ^1^H NMR (400 MHz, DMSO) *δ* 10.99 (1H, s), 8.23 (1H, s), 7.90 (1H, dd, *J* = 7.8, 1.9 Hz), 7.70–7.52 (1H, m), 7.42 (1H, dd, *J* = 7.1, 2.1 Hz), 7.39–7.28 (2H, m), 7.25 (1H, d, *J* = 8.4 Hz), 7.13 (1H, t, *J* = 7.5 Hz), 4.20 (2H, d, *J* = 7.0 Hz), 4.01 (3H, s), 2.29 (3H, s), 1.42 (1H, s). 13C NMR (101 MHz, DMSO) *δ* 177.91, 159.23, 157.47, 146.77, 146.74, 143.19, 142.86, 132.37, 127.56, 127.51, 126.28, 125.15, 120.76, 119.94, 115.11, 113.86, 113.74, 56.63, 55.74, 40.67, 40.46, 40.25, 40.04, 39.83, 39.62,39.42, 24.04, 16.59. HRMS calculated for [M + H], C_21_H_21_N_2_O_5_^+^*m*/*z*: 381.1445; found 381.14256.

#### 3.1.5 (*E*)-*N*′-[1-(8-Ethoxy-2-oxo-2*H*-chromen-3-yl)ethylidene]pyrazine-2-carbohydrazide (3e)

Yield 79%; UV-vis: *λ*_max_ = 426 nm (MeCN); FT-IR: 1577 cm^−1^ (C=N), 1673 cm^−1^ (C=O), 3308 cm^−1^ (NH); ^1^H NMR (400 MHz, DMSO) *δ* 11.08 (d, *J* = 7.9 Hz, 1H), 9.28 (s, 1H), 8.96 (s, 1H), 8.82 (1H, s), 8.26 (1H, s), 7.74–6.91 (3H, m), 5.02–3.51 (2H, m), 2.37 (2H, s), 1.43 (1H, s). 13C NMR (101 MHz, DMSO) *δ* 179.94, 159.39, 158.17, 148.17, 146.81, 143.27, 142.91, 129.54, 126.35, 125.20, 120.76, 119.86,115.20, 112.34, 112.11, 56.63, 40.66, 40.45, 40.24, 40.04, 39.83, 39.62, 39.41, 24.12, 16.96. HRMS calculated for [M + H], C_18_H_17_N_4_O_4_^+^*m*/*z*: 353.1244; found 353.12248.

#### 3.1.6 (*E*)-*N*′-[1-(8-Ethoxy-2-oxo-2*H*-chromen-3-yl)ethylidene]-1*H*-indole-3-carbohydrazide (3f)

Yield 75%; UV-vis: *λ*_max_ = 426 nm (MeCN); FT-IR: 1568 cm^−1^ (C=N), 1664 cm^−1^ (C=O), 3180 cm^−1^ (NH); ^1^H NMR (400 MHz, DMSO) *δ* 11.74 (1H, d, *J* = 3.0 Hz), 10.29 (1H, s), 8.44 (1H, s), 8.23 (2H, s), 7.55–7.44 (1H, m), 7.39 (1H, dd, *J* = 6.7, 2.5 Hz), 7.35–7.27 (2H, m), 7.17 (2H, pd, *J* = 7.1, 1.4 Hz), 4.20 (1H, d, *J* = 7.0 Hz), 2.32 (3H, s), 1.42 (2H, s). 13C NMR (101 MHz, DMSO) *δ* 177.49, 159.16, 157.89, 147.39, 146.78, 144.68, 143.20, 143.01, 138.88, 131.47, 131.30, 127.70, 127.59, 126.19, 125.18, 120.78, 119.89, 117.94, 115.18, 56.63,40.67, 40.46,40.26, 40.05, 39.84, 39.63, 39.42, 24.08, 16.66. HRMS calculated for [M + Na], C_22_H_20_N_3_O_4_^+^*m*/*z*: 390.1448; found 413.2665.

#### 3.1.7 (*E*)-*N*′-[1-(8-Ethoxy-2-oxo-2*H*-chromen-3-yl)ethylidene]furan-2-carbohydrazide (3g)

Yield 80%; UV-vis: *λ*_max_ = 426 nm (MeCN); FT-IR: 1578 cm^−1^ (C=N), 1692 cm^−1^ (C=O), 3339 cm^−1^ (NH); ^1^H NMR (400 MHz, DMSO) *δ* 10.67 (1H, s), 8.21 (1H, s), 7.95 (1H, dd, *J* = 1.7, 0.8 Hz), 7.40 (1H, dd, *J* = 7.2, 2.1 Hz), 7.35–7.26 (1H, m), 6.70 (1H, dd, *J* = 3.6, 1.7 Hz), 4.20 (1H, d, *J* = 7.0 Hz), 2.32 (2H, s), 1.42 (1H, s). 13C NMR (101 MHz, DMSO) *δ* 177.54, 159.16, 146.93, 146.77, 143.18, 142.94, 139.45, 128.66, 128.51, 126.19, 125.81, 125.68, 125.16, 120.77, 119.93, 115.14, 56.63, 40.67, 40.46, 40.25, 40.04, 39.83, 39.62, 39.41, 24.07, 16.59. HRMS calculated for [M + H], C_18_H_17_N_2_O_5_^+^*m*/*z*: 341.1132; found 341.1026.

#### 3.1.8 (*E*)-*N*-Benzyl-2-[1-(8-methoxy-2-oxo-2*H*-chromen-3-yl)ethylidene]hydrazine-1-carbothioamide (5a)

Yield 83%; UV-vis: *λ*_max_ = 426 nm (MeCN); FT-IR: 1541 cm^−1^ (C=N), 1180 cm^−1^ (C=S), 3292 cm^−1^ (NH); ^1^H NMR (400 MHz, DMSO) *δ* 10.56 (1H, s), 9.02 (1H, t, *J* = 6.2 Hz), 8.32 (1H, s), 7.40–7.27 (7H, m), 7.26–7.21 (1H, m), 4.86 (2H, d, *J* = 6.2 Hz), 3.92 (3H, s), 3.32 (2H, s), 2.28 (3H, s). 13C NMR (101 MHz, DMSO) *δ* 179.32, 159.28, 146.78, 146.68, 143.17, 142.51, 139.58, 128.65, 128.61, 127.90, 127.71, 127.24, 126.66, 125.16, 120.70, 119.86, 115.08, 56.62, 47.23, 40.67, 40.46, 40.25, 40.04, 39.83, 39.62, 39.42, 16.59. HRMS calculated for [M + H], C_20_H_20_N_3_O_3_S^+^*m*/*z*: 382.1220 found; 382.12006.

#### 3.1.9 (*E*)-*N*-(2,4-Dimethylphenyl)-2-[1-(8-methoxy-2-oxo-2*H*-chromen-3-yl)ethylidene] hydrazine-1-carbothioamide (5b)

Yield 84%; UV-vis: *λ*_max_ = 426 nm (MeCN); FT-IR: 1566 cm^−1^ (C=N), 1228 cm^−1^ (C=S), 3306 cm^−1^ (NH); 1H NMR (400 MHz, DMSO) *δ* 10.73 (1H, s), 9.80 (1H, s), 8.53 (1H, s), 7.46–7.23 (3H, m), 7.11 (3H, h, *J* = 4.6 Hz), 3.92 (3H, s), 2.34 (3H, s), 2.20 (6H, s). 13C NMR (101 MHz, DMSO) *δ* 177.23, 159.59, 159.10, 146.92, 146.77, 143.18, 142.95, 140.54, 129.39, 126.14, 125.15, 120.78, 119.92, 117.53, 115.15, 111.20, 111.01, 56.62, 55.65, 40.25, 40.04, 39.83, 16.55. HRMS calculated for [M + H], C_21_H_22_N_3_O_3_S^+^*m*/*z*: 396.1376; found 396.13576.

#### 3.1.10 (*E*)-2-[1-(8-Methoxy-2-oxo-2*H*-chromen-3-yl)ethylidene]-*N*-(4-methylbenzyl) hydrazine-1-carbothioamide (5c)

Yield 80%; UV-vis: *λ*_max_ = 426 nm (MeCN); FT-IR: 1569 cm^−1^ (C=N), 1214 cm^−1^ (C=S), 3283 cm^−1^ (NH); 1H NMR (400 MHz, DMSO) *δ* 10.53 (1H, s), 8.96 (1H, t, *J* = 6.2 Hz), 8.31 (1H, s), 7.31 (2H, s), 7.23 (2H, d, *J* = 7.8 Hz), 7.12 (2H, d, *J* = 7.8 Hz), 4.81 (2H, d, *J* = 6.1 Hz), 3.92 (3H, s), 2.27 (6H, d, *J* = 5.0 Hz). 13C NMR (101 MHz, DMSO) *δ* 179.19, 159.25, 146.77, 146.57, 143.16, 142.48, 136.49, 136.30, 129.20, 129.16, 127.94, 127.74, 126.64, 125.15, 120.69, 119.86, 115.06, 56.61, 47.01, 40.67, 40.46, 40.25, 40.04, 39.83, 39.63, 39.42, 21.16, 16.56. HRMS calculated for [M + H], C_21_H_22_N_3_O_3_S^+^*m*/*z*: 396.1376; found 396.13595.

#### 3.1.11 (*E*)-*N*-Isobutyl-2-[1-(8-methoxy-2-oxo-2*H*-chromen-3-yl)ethylidene]hydrazine-1-carbothioamide (5d)

Yield 82%; UV-vis: *λ*_max_ = 426 nm (MeCN); FT-IR: 1568 cm^−1^ (C=N), 1208 cm^−1^ (C=S), 3381 cm^−1^ (NH); 1H NMR (400 MHz, DMSO) *δ* 10.42 (1H, s), 8.48 (1H, t, *J* = 6.0 Hz), 8.30 (1H, s), 7.33 (3H, s), 3.93 (3H, s), 3.33 (2H, s), 2.27 (3H, s), 0.90 (6H, d, *J* = 6.7 Hz). 13C NMR (101 MHz, DMSO) *δ* 178.92, 159.02, 146.76, 145.77, 143.10, 142.40, 126.49, 125.13, 120.67, 119.88, 115.05, 56.61, 51.42, 40.66, 40.45, 40.24, 40.03, 39.82, 39.61, 39.40, 28.20, 20.55, 16.29. HRMS calculated for [M + H], C_17_H_22_N_3_O_3_S^+^*m*/*z*: 348.1376; found 348.13591.

#### 3.1.12 (*E*)-2-[1-(8-Methoxy-2-oxo-2*H*-chromen-3-yl)ethylidene]hydrazine-1-carbothioamide (5e)

Yield 84%; UV-vis: *λ*_max_ = 426 nm (MeCN); FT-IR: 1584 cm^−1^ (C=N), 1138 cm^−1^ (C=S), 3339 cm^−1^ (NH); 1H NMR (400 MHz, DMSO) *δ* 10.42 (1H, s), 8.44 (1H, s), 8.39 (1H, s), 7.95 (1H, s), 7.31 (2H, d, *J* = 1.7 Hz), 3.93 (3H, s), 2.27 (3H, s). 13C NMR (101 MHz, DMSO) *δ* 179.76, 159.29, 146.74, 146.38, 143.18, 142.63, 126.35, 125.13, 120.70, 119.97, 115.03, 56.61, 40.66, 40.45, 40.24, 40.03, 39.82, 39.61, 39.40, 16.46. HRMS calculated for [M + H], C_13_H_14_N_3_O_3_S^+^*m*/*z*: 292.0750; found 292.07348.

### 3.2. Biological activity

The antidiabetic potential of a novel class of chemicals 3(a–g) and 5(a–e) was assessed after they were synthesized. Table 1 lists the newly synthesized compound's IC_50_ and *r*^2^ values. Substituted hydrazides 2(a–g), thiosemicarbazides 5(a–e) and coumarin derivatives both served as exceptional structural frameworks, and the phenyl, indole and small heterocyclic group in hydrazide and thiosemicarbazide were crucial in creating a particular structure–activity relationship.

**Table 1:** Enzyme inhibition activity of 3a–g and 5a–e against α-glucosidase and α-amylase

Compound	IC_50_ (nM)		Metal chelating	Ki (nM)	Structure of compounds
	α-Glu	α-Amylase		α-Glu	
3a	325.73 ± 8.46	430.28 ± 7.60	>100	356.40 ± 11.73	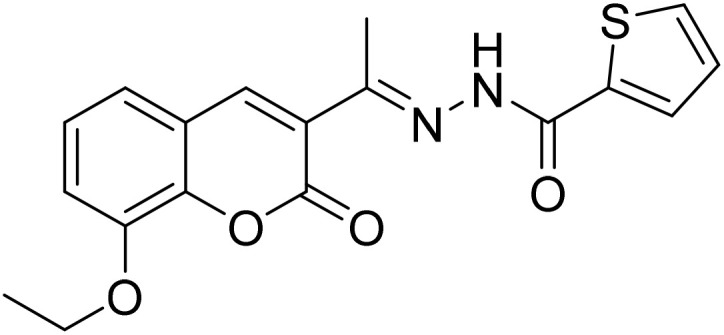
3b	252.45 ± 12.81	159.10 ± 8.15	>100	295.10 ± 13.45	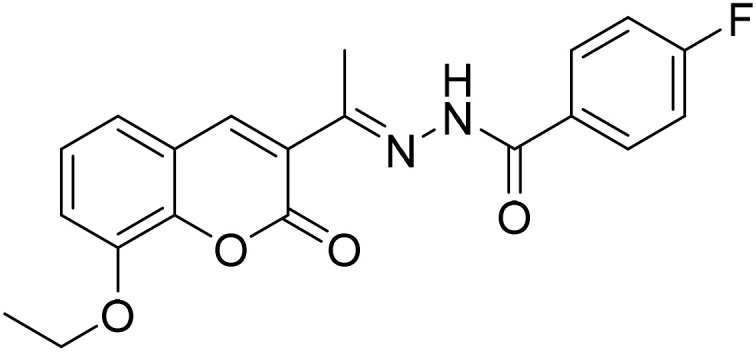
3c	295.08 ± 14.29	208.65 ± 12.40	>100	374.30 ± 12.70	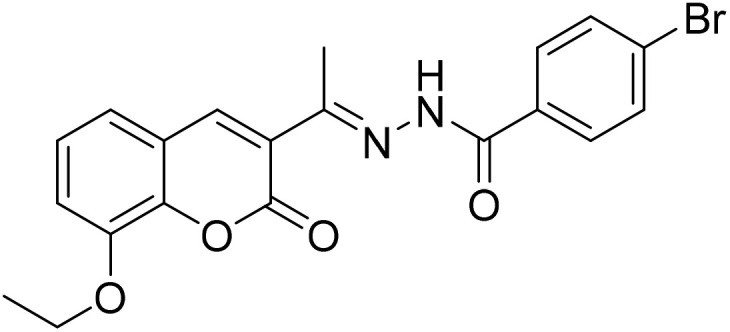
3d	275.17 ± 10.01	177.08 ± 8.25	>100	304.04 ± 10.42	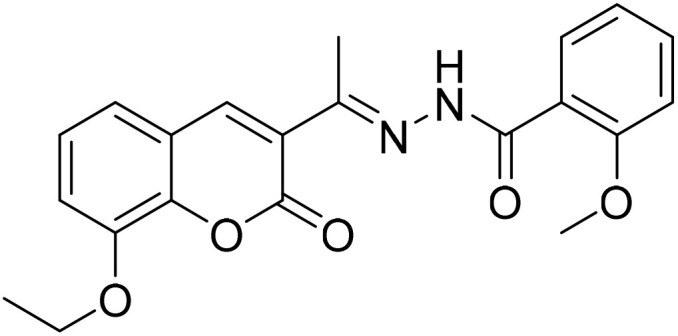
3e	307.35 ± 7.40	479.23 ± 8.70	>100	320.63 ± 15.83	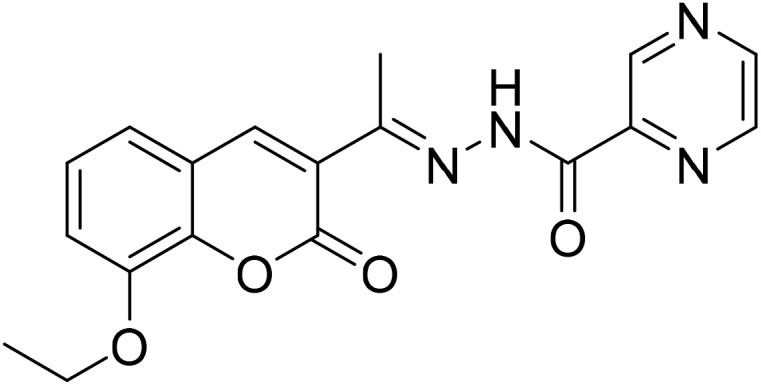
3f	318.26 ± 8.78	425.30 ± 11.08	>100	340.32 ± 9.31	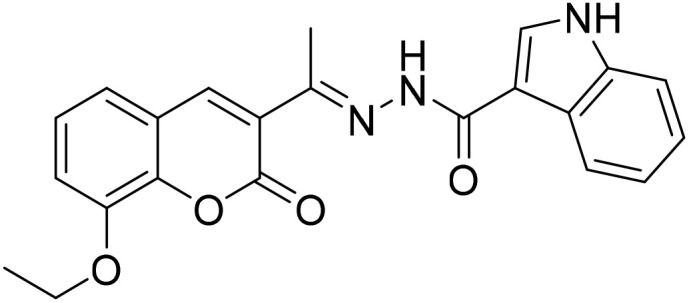
3g	260.63 ± 7.20	189.05 ± 9.65	>100	278.50 ± 8.15	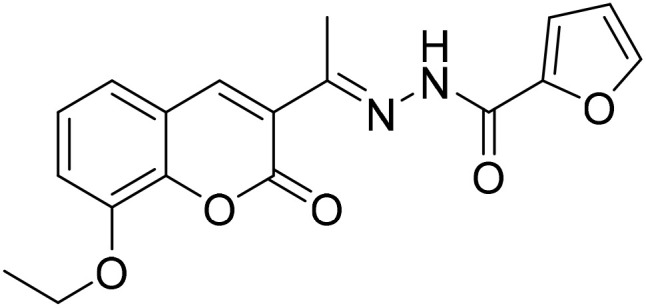
5a	189.73 ± 3.10	264.53 ± 2.64	>100	238.32 ± 8.37	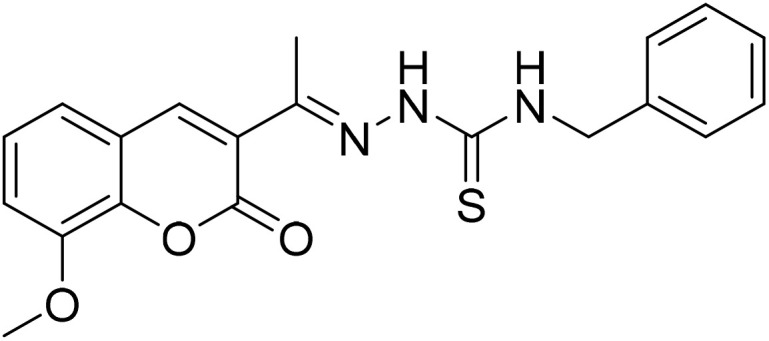
5b	110.45 ± 2.36	197.81 ± 6.04	58.06 ± 0.08	149.81 ± 3.24	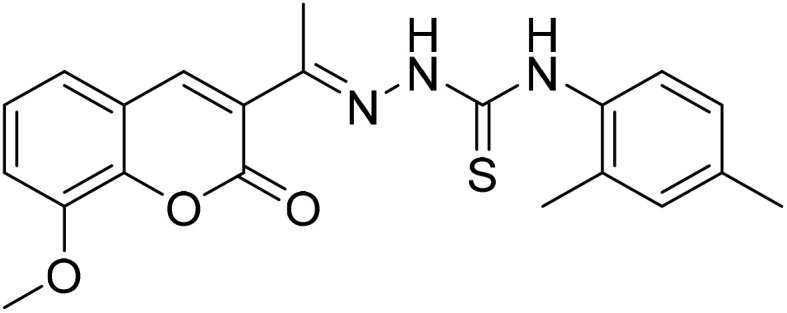
5c	88.04 ± 4.61	128.15 ± 2.82	43.55 ± 0.10	101.43 ± 5.75	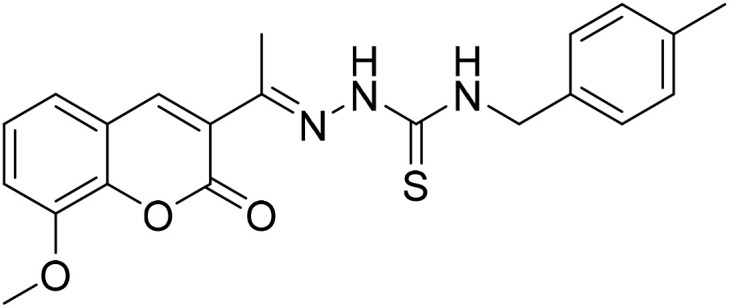
5d	164.27 ± 7.20	237.25 ± 3.43	50.38 ± 0.04	178.51 ± 4.67	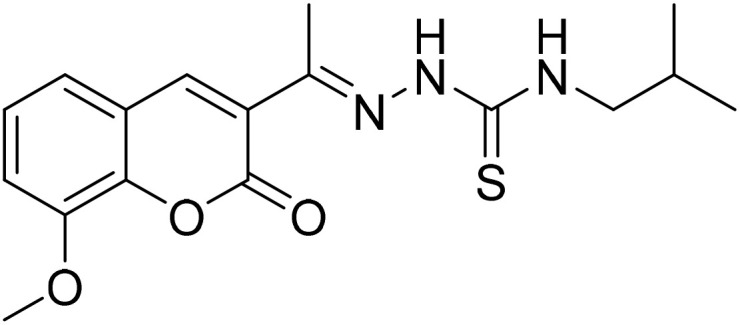
5e	73.68 ± 2.84	146.18 ± 7.35	35.96 ± 0.52	112.35 ± 3.80	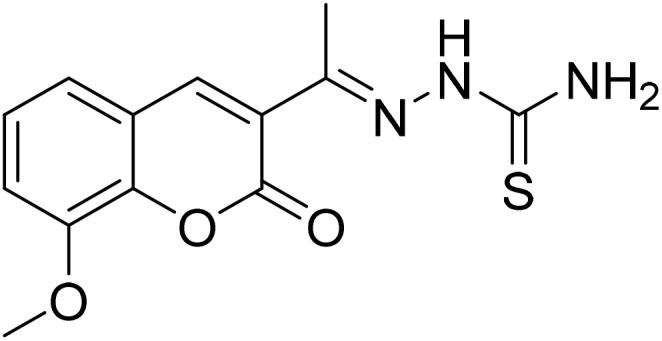
ACR	315.74 ± 15.06	437.93 ± 13.96	—	360.98 ± 11.38	Standard
EDTA	—	—	66.82 ± 0.40	—	Standard

### 3.3. Structure–activity relationship (SAR)

**Fig. 2:**
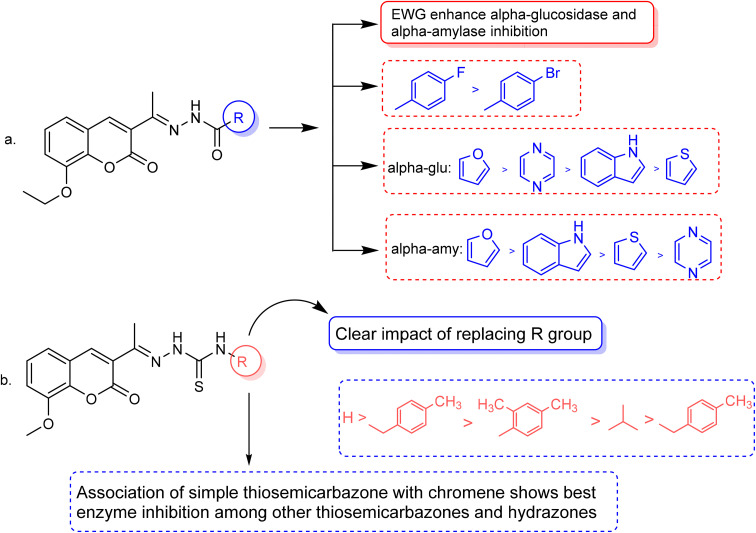
Structure–activity relationship (SAR) of the synthesized hydrazones (a) and thiosemicarbazones (b).

The inhibitory effects of chromone derivatives hydrazones (3a–3g) and thiosemicarbazones (5a–5e) on α-glucosidase and α-amylase have been investigated. Both sets demonstrated that structural changes maximize enzyme inhibition; some substances show more activity while others show less. Among the hydrazone series (Fig. 2a), compound 3b (4-F-phenyl hydrazone) is the most effective inhibitor of α-glucosidase and α-amylase, with IC_50_ values of 252.45 ± 12.81 nM and 159.10 ± 8.15 nM respectively. This is due to the presence of electron-withdrawing fluorine group on the phenyl ring, which improves the compound's enzyme active site interaction and inhibitory effectiveness. The furan-containing compound 3g is the second most effective hydrazone, with IC_50_ values of 260.63 ± 7.20 nM for α-glucosidase and 189.05 ± 9.65 nM for α-amylase. The furan ring, a tiny heterocyclic group, may create a favorable electrical environment for enzyme inhibition, making it one of the best performers in this series. Compound 3d, with a 2-OCH_3_-phenyl hydrazone group, has IC_50_ values of 275.17 ± 10.01 nM for α-glucosidase and 177.08 ± 8.25 nM for α-amylase. Despite higher α-amylase inhibition than α-glucosidase inhibition, the methoxy group on the phenyl ring may cause steric hindrance, resulting in a modest drop in potency as compared to 3b and 3g. Compound 3c, containing 4-Br-phenyl hydrazone, inhibits α-glucosidase moderately with IC_50_ values of 295.08 ± 14.29 nM and 208.65 ± 12.40 nM for α-amylase. The mild inhibition may be due to the bulky bromine group on the phenyl ring, which may hinder binding with enzyme's active site. Compound 3a, featuring a thiophene hydrazone group, demonstrates moderate inhibitory activity, with IC_50_ values of 325.73 ± 8.46 nM for α-glucosidase and 430.28 ± 7.60 nM for α-amylase. The higher IC_50_ values indicate that the thiophene group is less effective in enzyme binding than the furan and halogenated phenyl groups found in 3g and 3b, respectively. Compound 3f, featuring an indole group, exhibits IC_50_ values of 318.26 ± 8.78 nM for α-glucosidase and 425.30 ± 11.08 nM for α-amylase. Indole, while a biologically relevant scaffold, appears to exhibit lower enzyme inhibition compared to certain smaller heterocyclic or halogenated groups. The relatively larger structure may introduce steric hindrance, potentially reducing enzyme binding efficiency. Compound 3e, containing a pyrazole group, exhibits the lowest inhibition within the hydrazone series, presenting IC_50_ values of 307.35 ± 7.40 nM for α-glucosidase and 479.23 ± 8.70 nM for α-amylase.

In the thiosemicarbazone series (Fig. 2b), compound 6e, a simple thiosemicarbazone, exhibits the highest inhibitory potency, with IC_50_ values of 73.68 ± 2.84 nM for α-glucosidase and 146.18 ± 7.35 nM for α-amylase. The thiosemicarbazone's structure simplicity likely enhances enzyme binding through reduced steric hindrance, aligning with its greater inhibitory potency. Compound 5c, featuring a 4-methyl benzyl thiosemicarbazone moiety, demonstrates notable inhibitory activity, with IC_50_ values of 88.04 ± 4.61 nM for α-glucosidase and 128.15 ± 2.82 nM for α-amylase. The incorporation of a methyl group into the benzyl ring increases the compound's capacity to engage with the enzyme, thereby enhancing its potent inhibitory effects. Compound 5b, which contains a 2,4-dimethyl phenyl thiosemicarbazone group, exhibits IC_50_ values of 110.45 ± 2.36 nM for α-glucosidase and 197.81 ± 6.04 nM for α-amylase. The dimethyl substitution on the phenyl ring introduces steric bulk, potentially diminishing potency relative to simpler thiosemicarbazone compounds; however, it continues to demonstrate significant inhibition. Compound 5d, featuring an isobutyl thiosemicarbazone group, exhibits reduced inhibition, presenting IC_50_ values of 164.27 ± 7.20 nM for α-glucosidase and 237.25 ± 3.43 nM for α-amylase. Compound 5a, featuring a benzyl thiosemicarbazone moiety, exhibits the lowest inhibitory activity in this series, with IC_50_ values of 189.73 ± 3.10 nM for α-glucosidase and 264.53 ± 2.64 nM for α-amylase. The benzyl group offers less enhancement in enzyme's binding as compared to the simpler thiosemicarbazone or the more sterically optimized groups found in other compounds.

The study of hydrazone and the thiosemicarbazone series indicates that smaller, less sterically hindered structural motifs typically result in more effective enzyme inhibitors. In the hydrazone series, 3b and 3g exhibit the most potent inhibitory effects, with 3a following closely behind. The results underscore the significance of halogenation and heterocyclic groups in enhancing enzyme inhibition. Conversely, 3e (pyrazole) exhibits the lowest efficacy, as larger heterocycles or groups diminish potency due to steric hindrance. Within the thiosemicarbazone series, compounds 5e and 5c demonstrate the highest inhibitory activity, with 5e identified as the most potent compound overall. Simpler thiosemicarbazone structures exhibit superior performance attributed to diminish steric hindrance, facilitating more effective enzyme binding. Conversely, 5d and 5a demonstrate reduced activities, presumably attributable to the steric bulk of their side chains. These findings highlight the importance of structural simplicity and strategic substitution in enhancing enzyme inhibition, indicating that smaller, less bulky groups typically result in more effective inhibitors within both the hydrazone and thiosemicarbazone series.

### 3.4. Toxicity effects of best inhibitors (metal chelating)

In this section, we studied the metal chelating method, one of the antioxidant methods for toxicity. To stop the metal ions from serving as catalysts of lipid oxidation, metal-chelating antioxidants are thus added to food products. Ethylenediaminetetraacetic acid (EDTA) is now one of the most effective metal chelators utilized in the food business. In this study, EDTA was used as a standard. We used this method for all compounds, but the results were significant and good for the best inhibitors (Table 1). The results for the best inhibitors and for the standard were obtained as follows: 5e (IC_50_: 35.96 ± 0.52 μg mL^−1^) < 5c (IC_50_: 43.55 ± 0.10 μg mL^−1^) < 5d (IC_50_: 50.38 ± 0.04 μg mL^−1^) < 5b (IC_50_: 58.06 ± 0.08 μg mL^−1^) < EDTA (IC_50_: 66.82 ± 0.40 μg mL^−1^). In this part, the antioxidant values of the best inhibitors were good compared to the standard value EDTA (IC_50_: 66.82 ± 0.40 μg mL^−1^).

### 3.5. Computational studies

#### 3.5.1 Molecular docking simulations

**Fig. 3:**
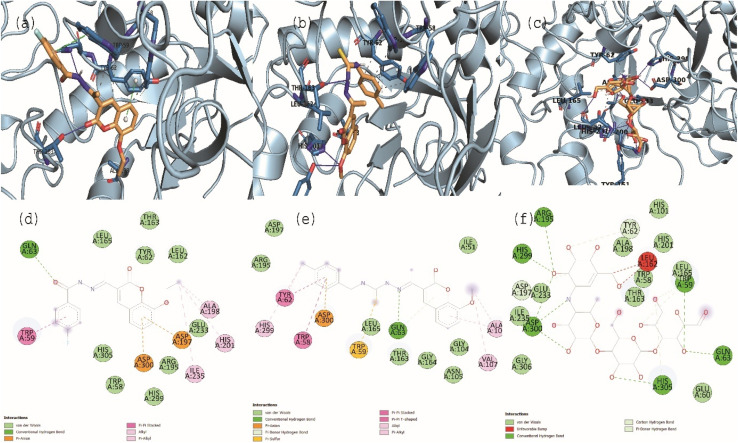
3D (Panel a to c) and 2D (Panel d to f) interaction diagrams for most active compounds 3b, 5c and acarbose, respectively against the target α-amylase.

**Fig. 4:**
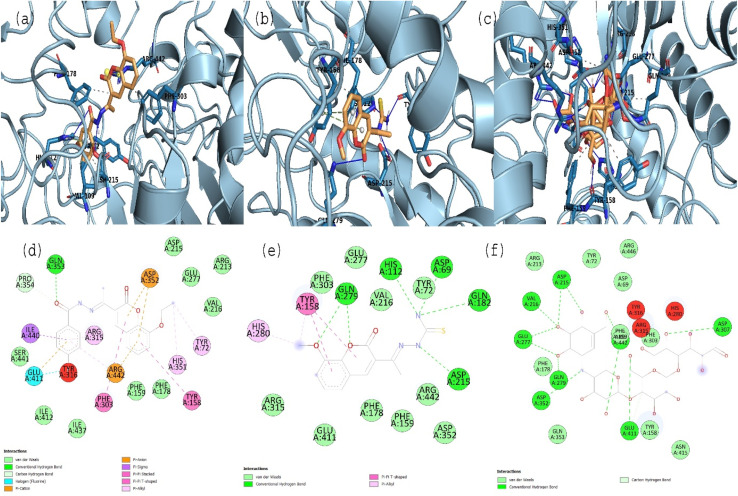
3D (Panel a to c) and 2D (Panel d to f) interaction diagrams for most active compounds 3b, 5e and acarbose, respectively against the target α-glucosidase.

Synthesized candidates were subjected for *in silico* analysis using ‘molecular docking’ to see potential interaction against the selected targets, *i.e.*, α-glucosidase (PDB: 3A4A) and α-amylase (PDB: 3BAJ) (Fig. 3 and 4). The molecular docking analysis was carried out using the ‘Autodock V. 2’. Among a carbohydrazide series, molecule 3b was obtained as best docked candidate both in *in silico* and *in vitro* analysis. Compound 3b was interacted with target α-glucosidase (PDB: 3A4A) (docking score: −7.23 kcal mol^−1^) *via* amino acid residues such as Gln353 (H-bonding–C=O–), Ile440 (π–Sigma interactions), Glu411 (π–anionic interactions), Phe303 (π–Sigma interactions), Tyr158 (π–Sigma interactions), His351 (π–π interactions), Tyr72 (π–π interactions), and Asp352 (π–anionic interactions) (Fig. 3d). While, compound 3b had interactions with target α-amylase (PDB: 3BAJ) (docking score: −7.68 kcal mol^−1^) *via* Gln63 (conventional H-bond–C=O–), Trp59 (π–π aryl), Asp300 (π–anionic interaction), Asp197 (π–anionic interaction), Ile235 (alkyl), Ala198 (alkyl–alkyl interactions), and His201 (mainly hydrophobic) (Fig. 3d).

In case of carbothioamide series, compound 5c have obtained higher affinity towards target α-amylase (docking score: −9.36 kcal mol^−1^), while compound 5e had highest docking score (docking score: −8.55 kcal mol^−1^) against α-glucosidase target than other compounds. Compound 5e interacted with His280 (hydrophobic), Tyr158 (hydrophobic), Gln279 (H-bonding), His112 (H-bonding), Asp69 and Asp215 (H-bonding) residues of binding cavity of α-glucosidase (Fig. 3e). In case of binding of 5c towards α-amylase, we observed residues His299 (alkyl interactions), Tyr62 (π–π stacking), Trp58 (π–π stacking), Asp300 (π–anionic), Trp59 (π–anionic–C=S–moiety), Leu165, Gln63 (H-bonding with –N=-moiety), and Val107 (hydrophobic) in the binding site (Fig. 3e). For both enzymes, the standard acarbose retained comparatively lower docking score as compared to that of carbohydrazide and carbothioamide compounds (docking score for acarbose: −6.73 and −7.23 kcal mol^−1^) against α-glucosidase (PDB: 3A4A) and α-amylase (PDB: 3BAJ), respectively. Our results obtained from *in silico* are comparable to actual *in vitro* data indicating the more insights into binding characteristics of novel compounds.

#### 3.5.2 *In silico* ADME analysis

The *in silico* ADME (Absorption, Distribution, Metabolism, and Excretion) profile of molecules 3a to 5e reveals promising characteristics for drug development (Table 2). All compounds demonstrate high gastrointestinal absorption, ensuring efficient oral bioavailability. However, none of the molecules are permeable to the blood–brain barrier (BBB), making them suitable for peripheral targets rather than central nervous system disorders. Additionally, none are substrates for P-glycoprotein (Pgp), which enhances their systemic availability. All compounds inhibit key cytochrome P450 enzymes, including CYP1A2, CYP2C19, CYP2C9, and CYP3A4, suggesting potential drug–drug interactions. Importantly, no inhibition of CYP2D6 was observed, minimizing interference with certain psychotropic drugs. The log *K*_p_ values suggest moderate permeability across biological membranes, while adherence to Lipinski's Rule of Five supports good oral bioavailability. Synthetic accessibility scores indicate moderate ease of synthesis, making these molecules viable for further development, although metabolic interactions require careful consideration. Overall, these molecules showed favorable pharmacokinetic properties for peripheral drug targets.

**Table 2:** *In silico* analysis of ADME properties of synthesized molecules

Molecule	GI absorption	BBB permeant	Pgp substrate	CYP1A2 inhibitor	CYP2C19 inhibitor	CYP2C9 inhibitor	CYP2D6 inhibitor	CYP3A4 inhibitor	Log *K*_p_ (cm s^−1^)	Lipinski #violations	Bioavailability score	Synthetic accessibility
3a	High	No	No	Yes	Yes	Yes	No	Yes	−5.92	0	0.55	3.47
3b	High	No	No	Yes	Yes	Yes	No	Yes	−5.88	0	0.55	3.52
3c	High	No	No	Yes	Yes	Yes	No	Yes	−5.88	0	0.55	3.52
3d	High	No	No	Yes	Yes	Yes	No	Yes	−6.09	0	0.55	3.68
3e	High	No	No	Yes	No	Yes	No	Yes	−7.19	0	0.55	3.41
3f	High	No	No	Yes	Yes	Yes	No	Yes	−6.04	0	0.55	3.56
3g	High	No	No	Yes	Yes	Yes	No	Yes	−6.25	0	0.55	3.54
5a	High	No	No	Yes	Yes	Yes	No	Yes	−6.15	0	0.55	3.63
5b	High	No	No	No	Yes	Yes	No	Yes	−5.67	0	0.55	3.78
5c	High	No	No	Yes	Yes	Yes	No	Yes	−6.06	0	0.55	3.64
5d	High	No	No	Yes	Yes	Yes	No	Yes	−6.06	0	0.55	3.64
5e	High	No	No	No	No	No	No	No	−6.95	0	0.55	3.25

#### 3.5.3 DFT calculation

**Fig. 5:**
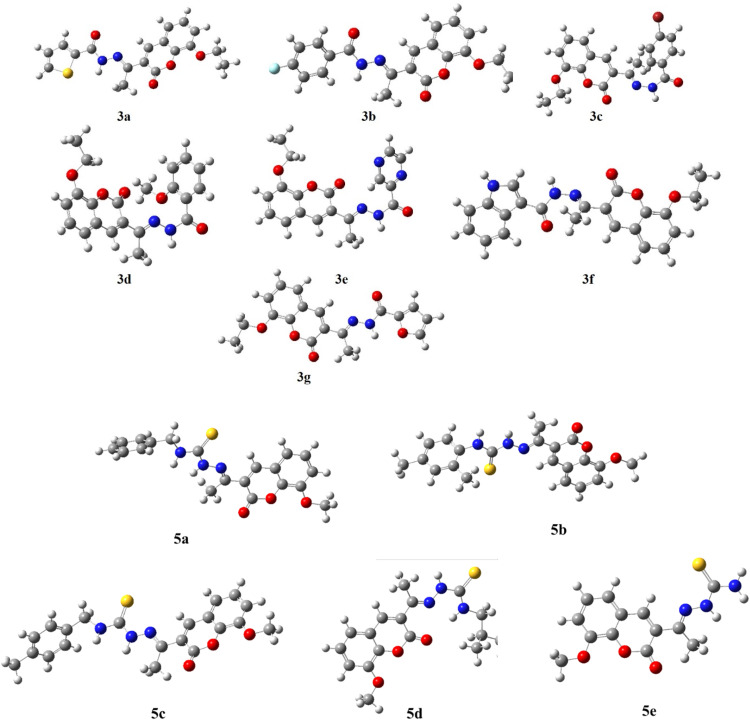
Energy optimized structures of all compounds by using B3LYP/6-311+G (d, p) 3(a–g) and 5(a–e).

DFT is a potent computational method that provides a thorough understanding of molecule's electrostatic environment, facilitating understanding of reactivity, non-covalent interactions, and other molecular characteristics. It has important uses in materials research, drug development, and other domains where knowledge of molecular interactions is crucial. The optimized geometry of synthesized compounds is given in Fig. 5. HOMO–LUMO transitions were used to assess the molecular interactions and binding affinities of synthetic molecules inside a biological system. The ionization potential, or the capacity of molecules to donate electrons, is indicated by the highest occupied molecular orbital (HOMO) in the framework of frontier molecular orbitals (FMO), whereas the electron affinity, or the capacity of molecules to accept electrons, is indicated by the lowest unoccupied molecular orbital (LUMO). Two important quantum chemical parameters that describe a molecule's reactivity, shape, and binding characteristics as well as the behavior of its alternatives and fragments are its HOMO and LUMO energy levels. The reactivity and kinetic stability of the molecule are determined by the energy differential between HOMO and LUMO. A molecule with a lower energy gap (Δ*E*_gap_) between HOMO and LUMO is more likely to have a better affinity for the targeted receptor enzyme and to be capable of intramolecular charge transfer, both of which lead to increased biological activity. While molecules with a short HOMO–LUMO gap are more reactive, less stable, and categorized as soft, those with a big gap are less polarizable, less reactive, and classified as hard.

**Fig. 6:**
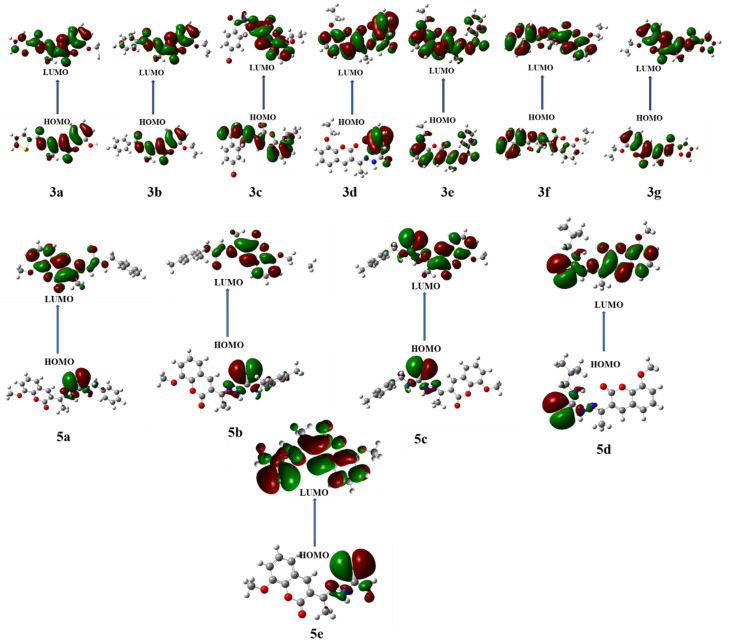
HOMO–LOMO energy gap of 3(a–g) and 5(a–e).

When a compound has a small Δ*E* value, a noticeable intramolecular charge transfer from an electron donor to an electron acceptor moiety is observed. With a lower Δ*E* value, the molecule is generally less stable and more reactive. The experimentally hit compound's HOMO–LUMO contour plots are shown in Fig. 6. The HOMO–LUMO Δ*E* values and other global reactivity parameters are given in Table 3. The lowest value of Δ*E* (0.1285 and 0.1117 eV) is observed with the compound 3b and 5e. Thus, these compounds (3b and 5e) are predicted to be the most reactive ones as matched with experimentally work. On the flip side of it, 3e and 3f compound exhibited the highest value of Δ*E* = 0.1429 and 0.1427 eV, which in turn is found to be the most stable one than all other compounds studied.

**Table 3:** HOMO–LUMO and other global reactivity parameters

Code	HOMO	LUMO	Δ*E* = *E*_LUMO_ − *E*_HOMO_	(*μ*)	(*η*)	*χ*	*ω*
3a	0.23009	−0.09195	0.13814	−0.02288	0.184115	0.01144	0.000261747
3b	−0.2209	−0.0924	0.1285	−0.03815	0.1747	0.014075	0.000396211
3c	−0.2424	−0.1008	0.1416	−0.0301	0.192	0.015	0.00045
3d	−0.2335	−0.0937	0.1398	−0.0238	0.18665	0.0119	0.00028322
3e	−0.2407	−0.0978	0.1429	−0.02635	0.1918	0.013175	0.000347161
3f	−0.2213	−0.0786	0.1427	−0.0325	0.182	0.003625	2.62813 × 10^−5^
3g	−0.2279	−0.0899	0.138	−0.0209	0.18295	0.01045	0.000218405
5a	−0.2071	−0.088	0.1191	−0.02845	0.1631	0.014225	0.000404701
5b	−0.2036	−0.0884	0.1152	−0.0308	0.1594	0.0154	0.00047432
5c	−0.2056	−0.0873	0.1183	−0.02815	0.16195	0.014075	0.000396211
5d	−0.21	−0.0912	0.1188	−0.0318	0.1644	0.0159	0.00050562
5e	−0.2083	−0.0966	0.1117	−0.04075	0.16	0.020375	0.000830281

In addition to this, other parameters such as chemical potential (*μ*) electronegativity (*χ*), global electrophilicity index (*ω*) and chemical hardness (*η*) are computed by HOMO–LUMO energy level by using equation given below. The compounds 3b, 5a and 5e have the lowest value of hardness which means they are more reactive. In case of hydrazone and thiosemicarbazone, the compounds, 3b and 5e were observed as more reactive among all investigated compounds due to the highest value of chemical potential (−0.03815 and −0.04075 eV) because the high value of chemical potential is linked with the high reactivity and least stability of a compound.1
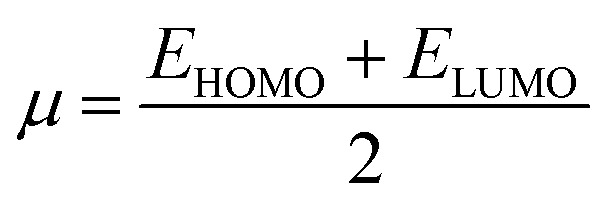
2
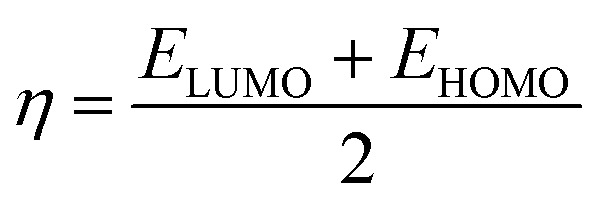
3
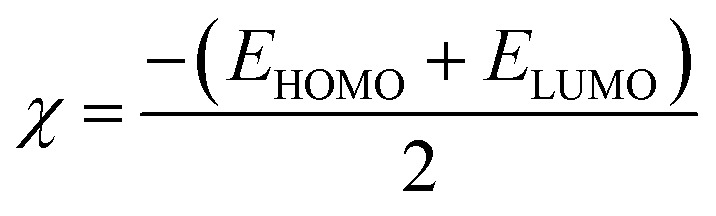
4
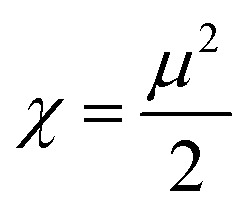


##### 3.5.3.1. Molecular electrostatic potential (MEP)

**Fig. 7:**
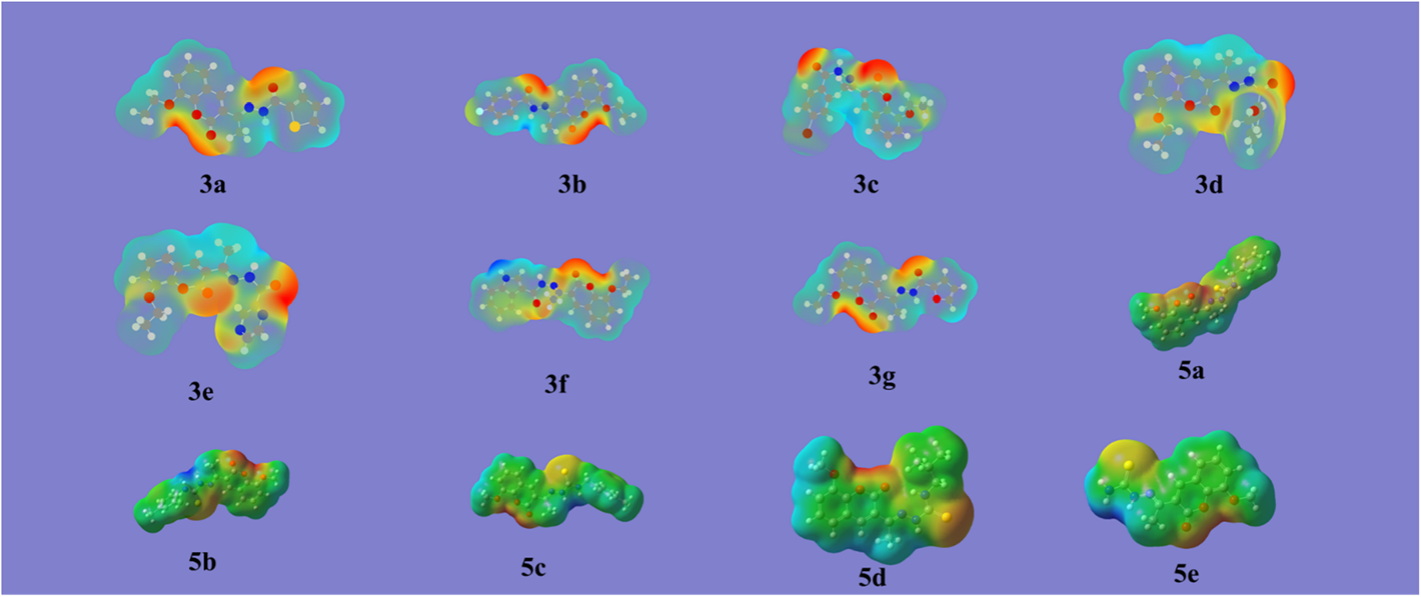
Molecular electrostatic potential (MEP) of all compounds.

Maps of molecular electrostatic potential (MEP) show the reactive and interaction sites in molecules, giving information about their size, structure, and areas that are vulnerable to nucleophilic and electrophilic attacks. These maps facilitate comprehension of chemical reaction pathways by illustrating electrostatic potential. Fig. 7 showed the calculated map for 3(a–g) and 5(a–e) series.

When looking at the MEP maps, one can see that the green areas are neutral regions and the blue areas are the compound's N–H groups, which are suitable for nucleophilic attacks because they are electron-donating sites. In general, oxygen atoms are highlighted in red and yellow, indicating electron-withdrawing sites that are advantageous for electrophilic attacks. Thus, the most reactive areas are the red ones. Interestingly, the electrical characteristics of the produced compounds are not greatly affected by substituent variations. This implies that the main binding sites in synthetic structures are made up of coumarin derivatives are nitrogen and oxygen atoms. The binding mechanisms with biological receptors can be interpreted similarly. Yellow areas are home to functional groups including methyl, bromine, fluorine, and methoxy.

##### 3.5.3.2. Non-covalent interaction/RDG/NCI

**Fig. 8:**
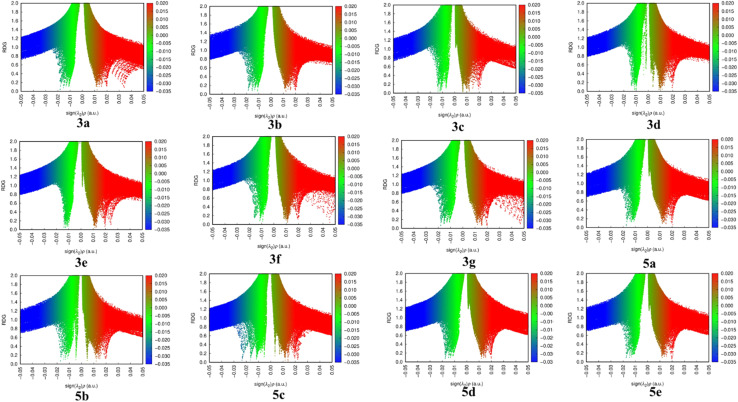
RDG plot of 3(a–g) and 5(a–e).

The non-covalent interactions found in the synthesized 3(a–g) and 5(a–e) series were investigated using the Multiwfn software, and the images produced were displayed using VMD and irfan view software. The RDG v/s sign of (*λ*_2_) × *ρ* was plotted in Fig. 8 to show the 2D and 3D iso surfaces of the produced molecules. The weak interactions between and within the molecules that result from the quantum-mechanical electron density have been examined using Reduced Density Gradient (RDG) studies. One essential dimensionless quantity in RDG that tells us about the strength of the interactions is the electron density value. The definition of RDG isRDG(*r*) = |Δ*ρ*(*r*)|/2(3·π·2)1/3*ρ*(*r*)4/3In this case, *ρ*(*r*) represents the electron density, Δ*ρ*(*r*) represents the electron density norm, and the eigen value is a sign of (*λ*_2_). The van der Waals interaction is shown as green in Fig. 8, whereas red denotes a strong repulsion and blue denotes a strong attractive interaction. In addition to the 2D plots of RDG that show hydrogen bonding from 0.05 to 0.02 a.u., the peaks from 0.02 to +0.01 a.u. indicate the presence of non-covalent contact and 0.02 to 0.05 a.u. indicate high repulsion between the molecule's atoms. The 3D graph shows that the synthesized compounds exhibit strong repulsive contact.

## 4. Conclusion

The synthesized compounds exhibit the potent potential of inhibition against α-amylase, and α-glucosidase. The compound 3b from carbohydrazide series, demonstrated the strongest potency against α-amylase and α-glucosidase, with respective IC_50_ values of 252.45 ± 12.81 nM and 159.10 ± 8.15 nM and in case of carbothioamide series, compound 5e, a simple thiosemicarbazone, exhibits the highest inhibitory potency, with IC_50_ values of 73.68 ± 2.84 nM for α-glucosidase and 146.18 ± 7.35 nM for α-amylase, which attribute to the presence of strong electron withdrawing substituents. These findings are further confirmed by the *in silico* studies demonstrating substantial binding affinities of ligands 3b, and 6e with target proteins (Acarbose) having docking scores of −7.23 to −8.55 kcal mol^−1^. The analyzed coumarin derivatives demonstrated drug-like properties, physiochemical characteristics and ADME results showed good intestinal absorption. The DFT studies revealed low energy gap (0.1285 and 0.1117 eV) and lowest hardness values of 0.1747 and 0.1600 reflecting the high reactivity of top hits. Furthermore, the electrostatic potential distribution on the molecule surface to support their interactions with important amino acid residues was disclosed by MEP analysis. According to these results, the top hit compounds could be considered as prospective therapeutic candidates for the management and treatment of diabetes mellitus. Additionally, the antioxidant (metal chelating) values of the best inhibitors were good compared to the standard value EDTA (IC_50_: 66.82 ± 0.40 μg mL^−1^).

## Author contributions

Wajeeha Zareen, Mussarat Tasleem: investigation, formal analysis. Nadeem Ahmed: writing – original draft, validation, data curation. Suraj N. Mali, Abdullah K. Alanazi, Naflaa A. Aldawsari: computational studies, software, data curation, resources. Nastaran Sadeghian, Parham Taslimi: biological assays, software, formal analysis. Muhammad Tahir: DFT studies, data curation. Zahid Shafiq: writing – original draft, supervision, conceptualization. Ali Muhammad Khan: supervision, conceptualization.

## Conflicts of interest

The authors declare that they have no known competing financial interests or personal relationships that could have appeared to influence the work reported in this paper.

## Supplementary Material

RA-015-D5RA04619J-s001

## Data Availability

The data used for the manuscript will be included in an supplementary information file (SI), available online on *RSC Advances* web site. Supplementary information is available. See DOI: https://doi.org/10.1039/d5ra04619j.
