# Supplementary material for: Synthesis, antidiabetic evaluation, and computational modeling of 3-acetyl-8-ethoxy coumarin derived hydrazones and thiosemicarbazones
Source: RSC Adv. 2025 Oct 16;15(46):39043–58. doi: 10.1039/d5ra04619j (PMC12530850; doi:10.1039/d5ra04619j)

Supplementary Information (SI) for RSC Advances.
This journal is © The Royal Society of Chemistry 2025

Supplementary Information File

H[1] & ^{13}CNMR Spectrum of Compound 3a

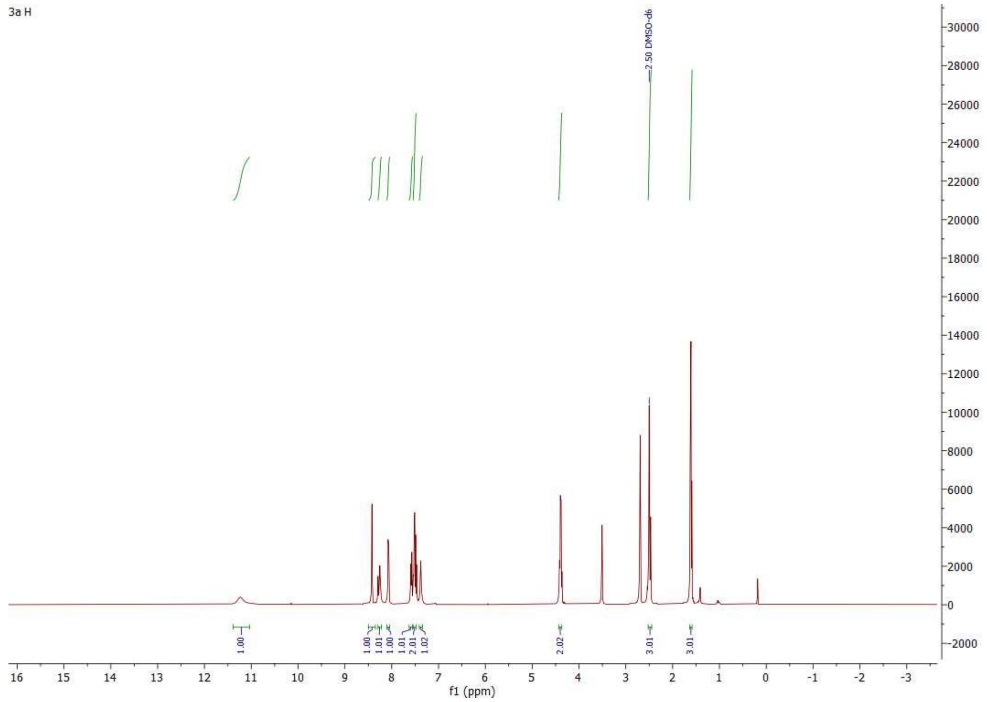

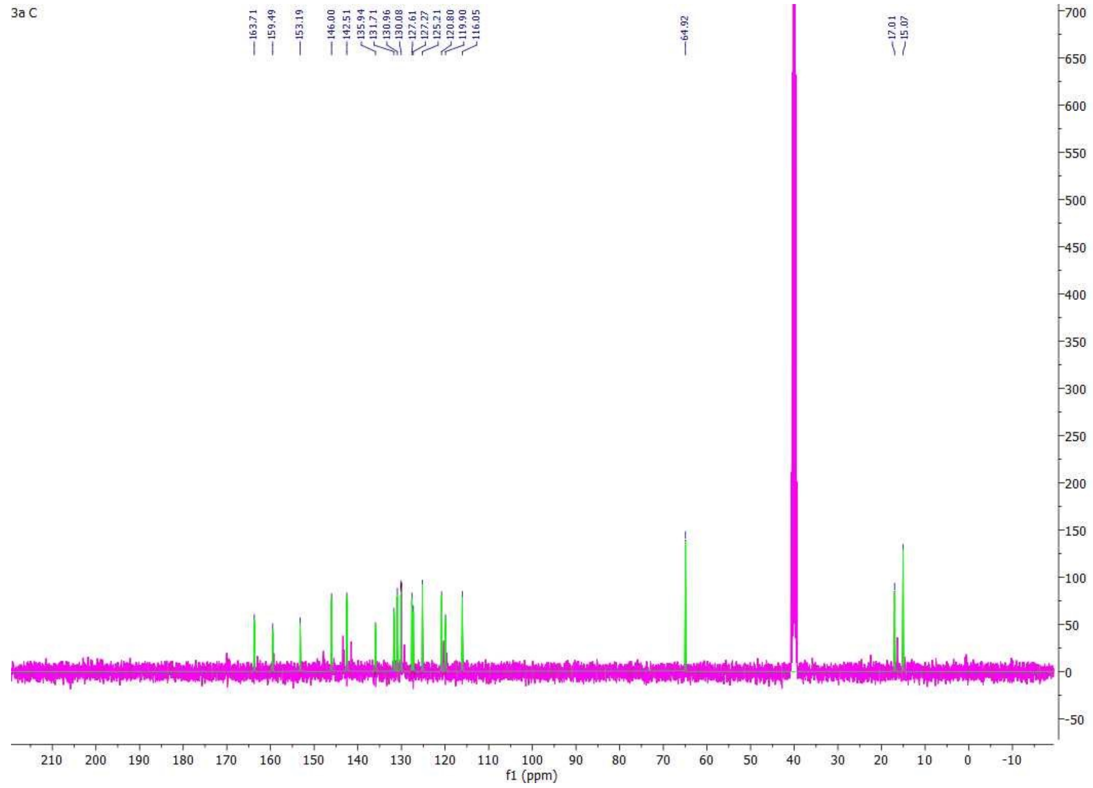

H^1 & ^{13}CNMR Spectrum of Compound 3b

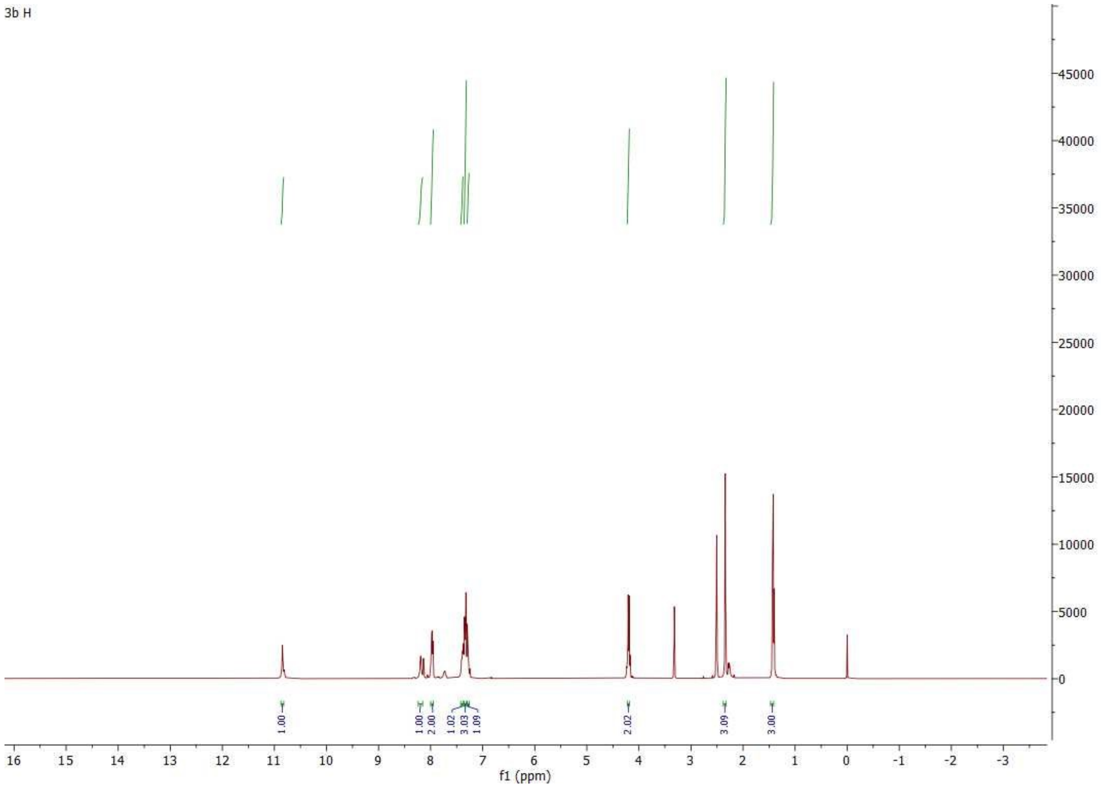

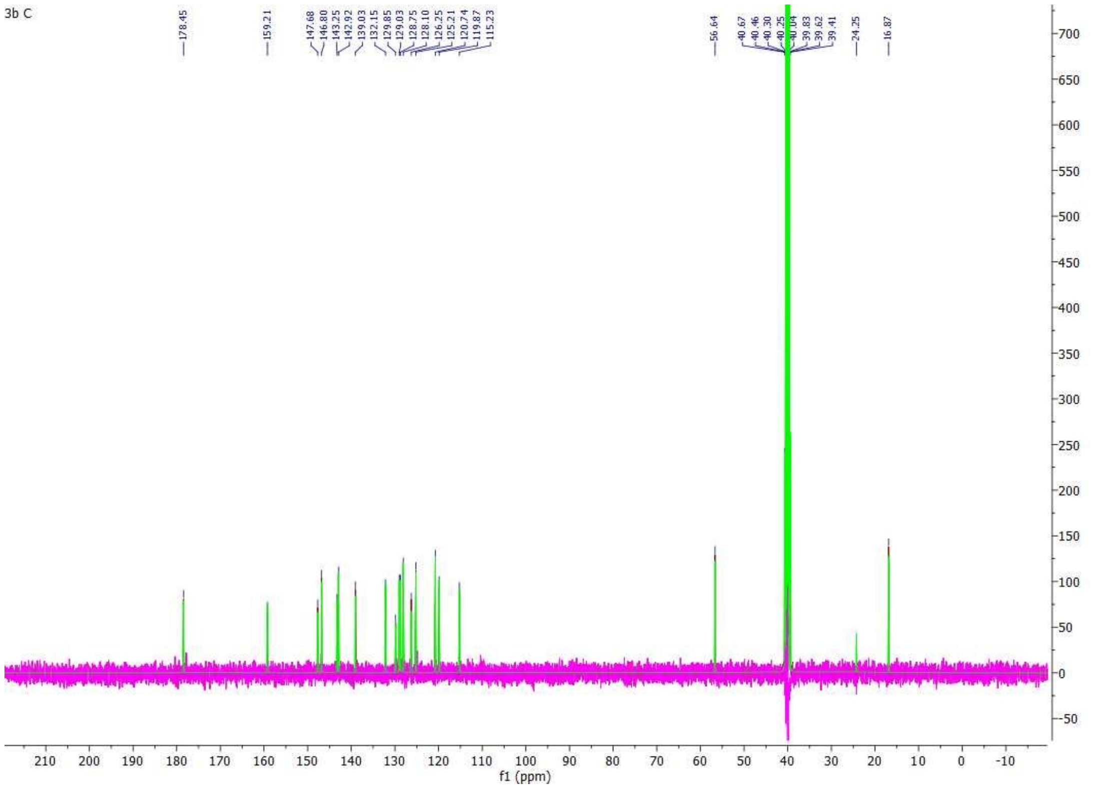

H[1] & ^{13}CNMR Spectrum of Compound 3c

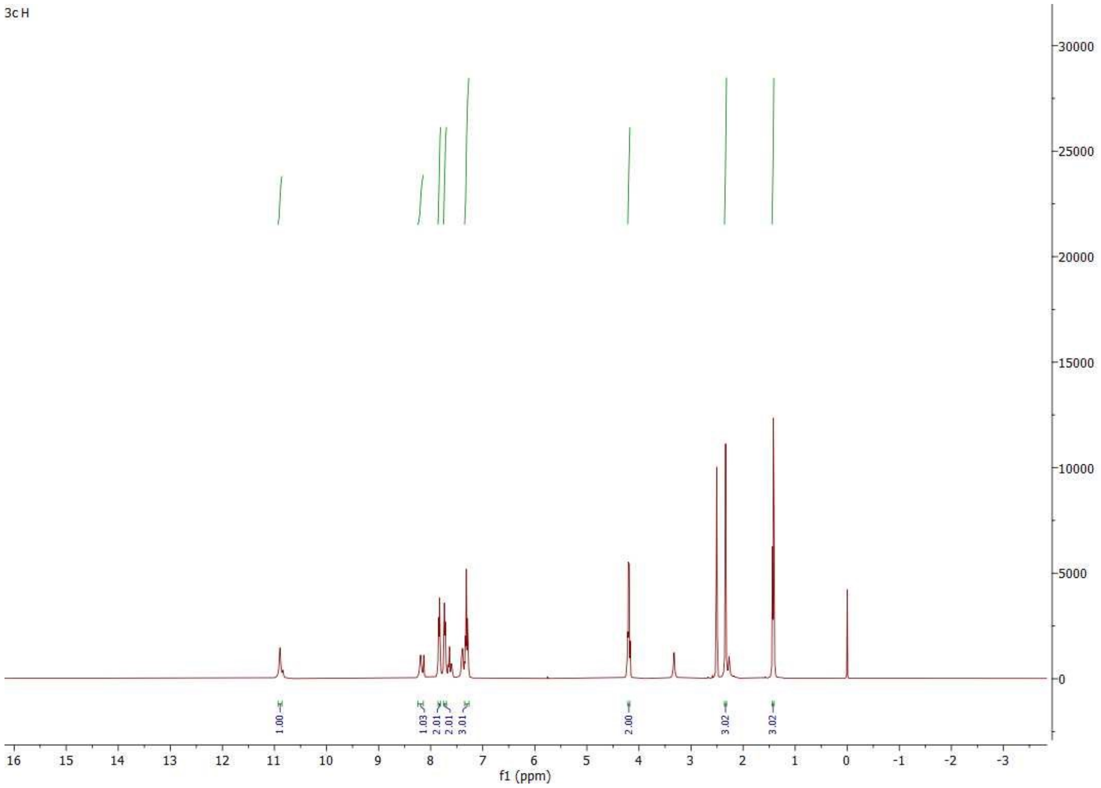

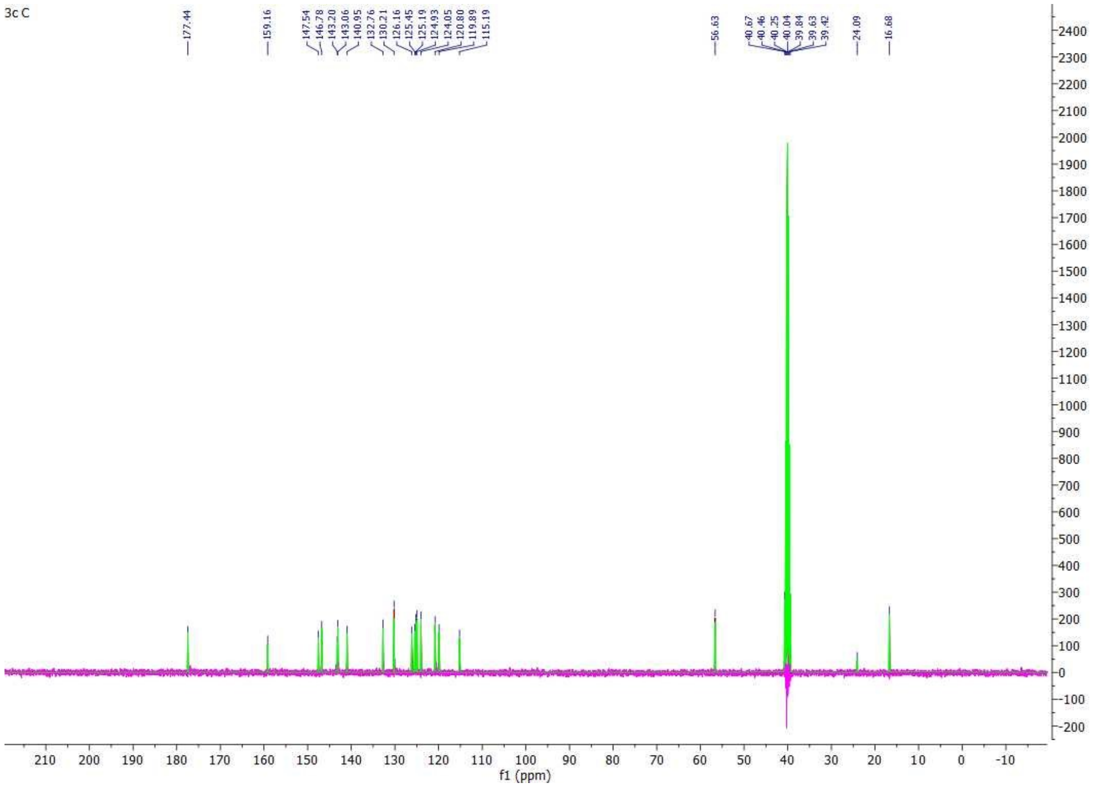

H[1] & ^{13}CNMR Spectrum of Compound 3d

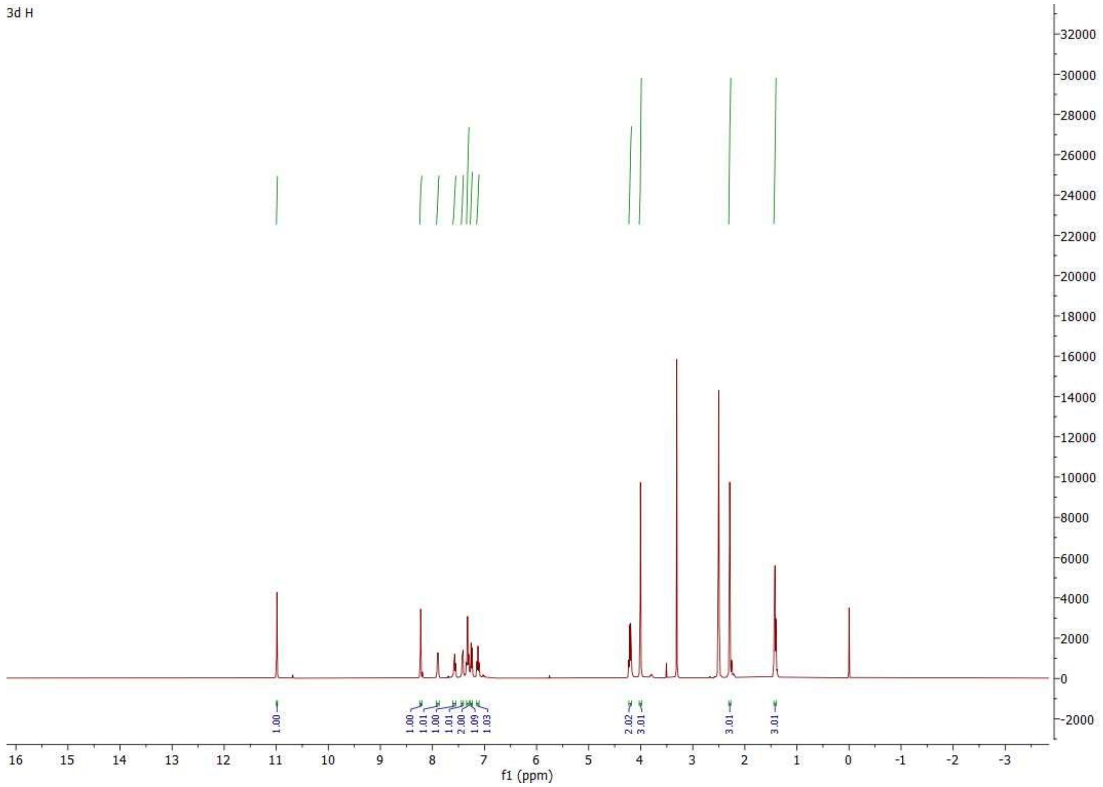

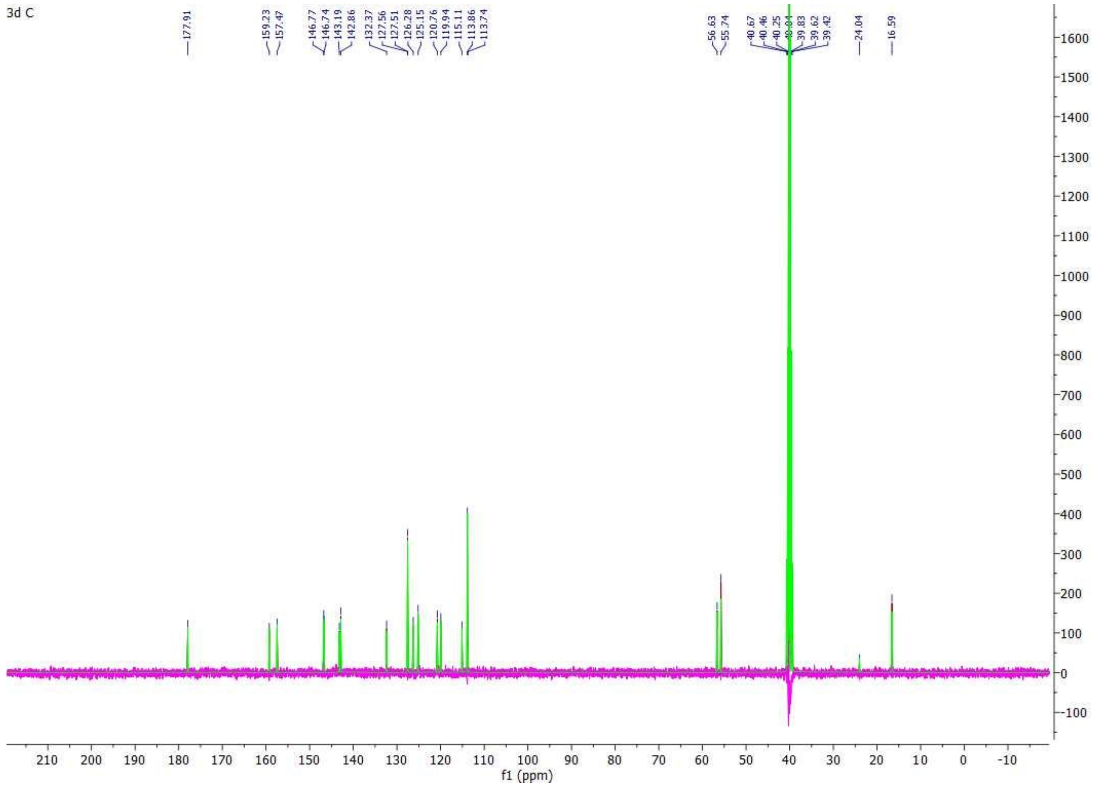

H[1] & ^{13}CNMR Spectrum of Compound 3e

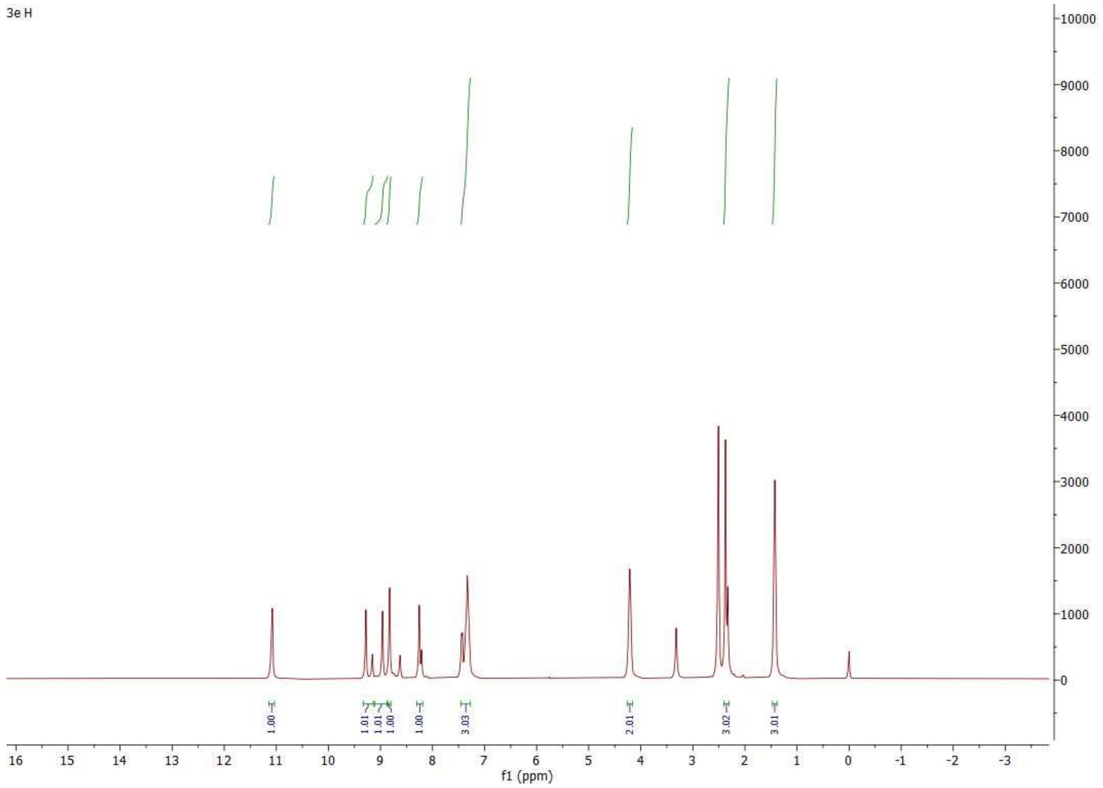

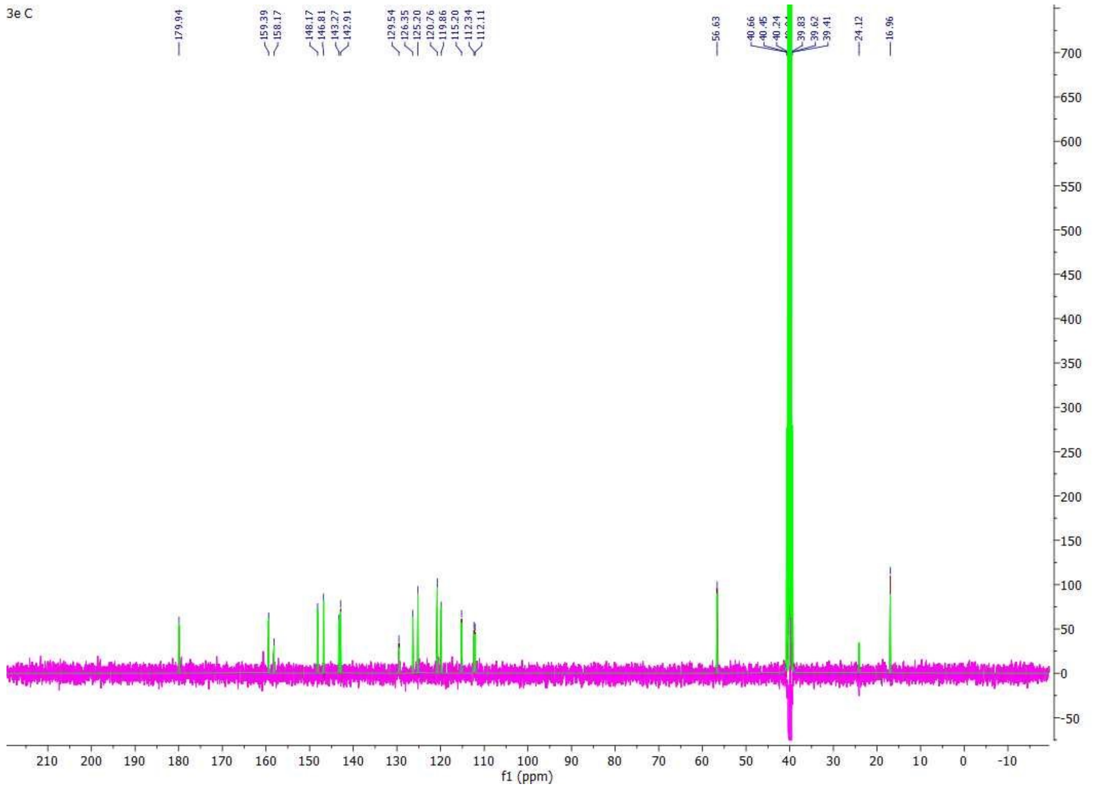

H^1 & ^{13}CNMR Spectrum of Compound 3f

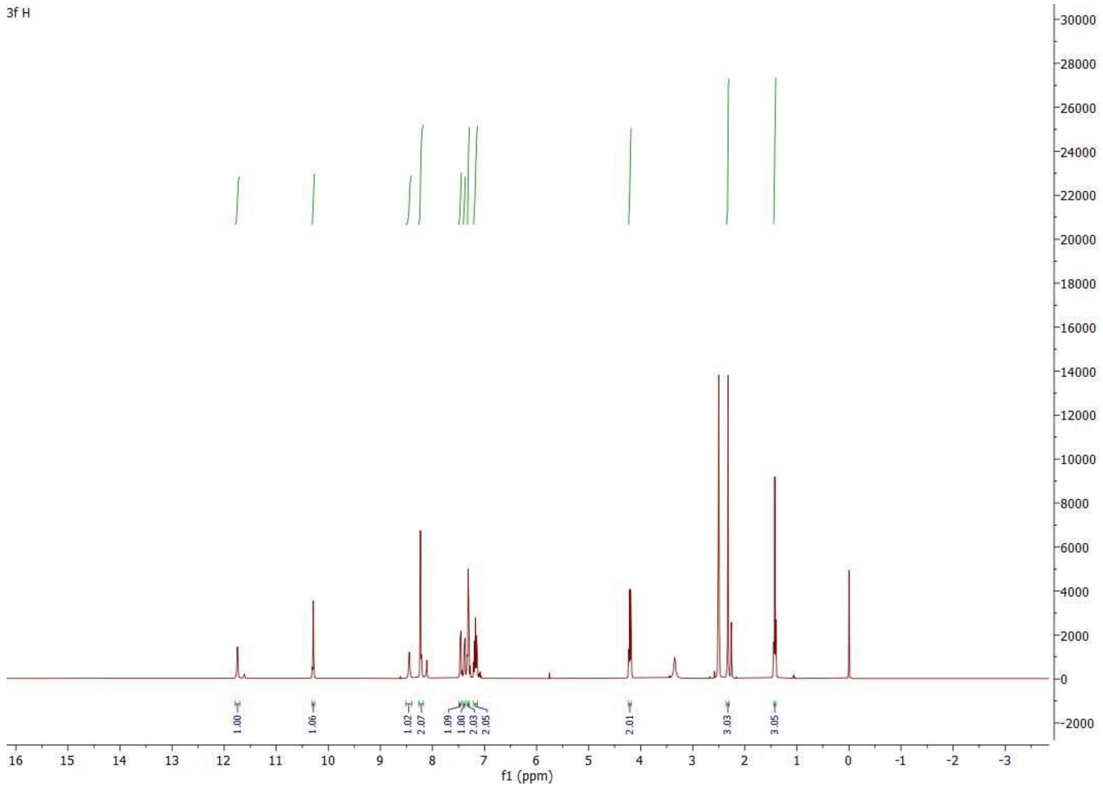

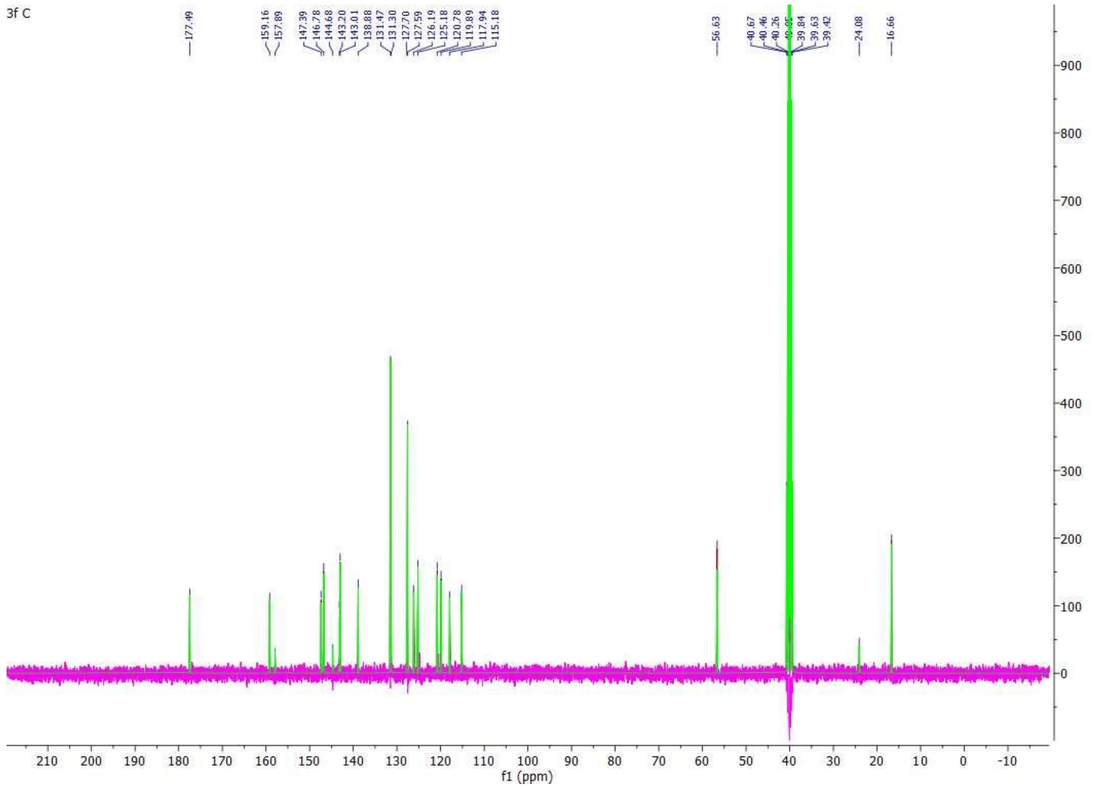

H[1] & ^{13}CNMR Spectrum of Compound 3g

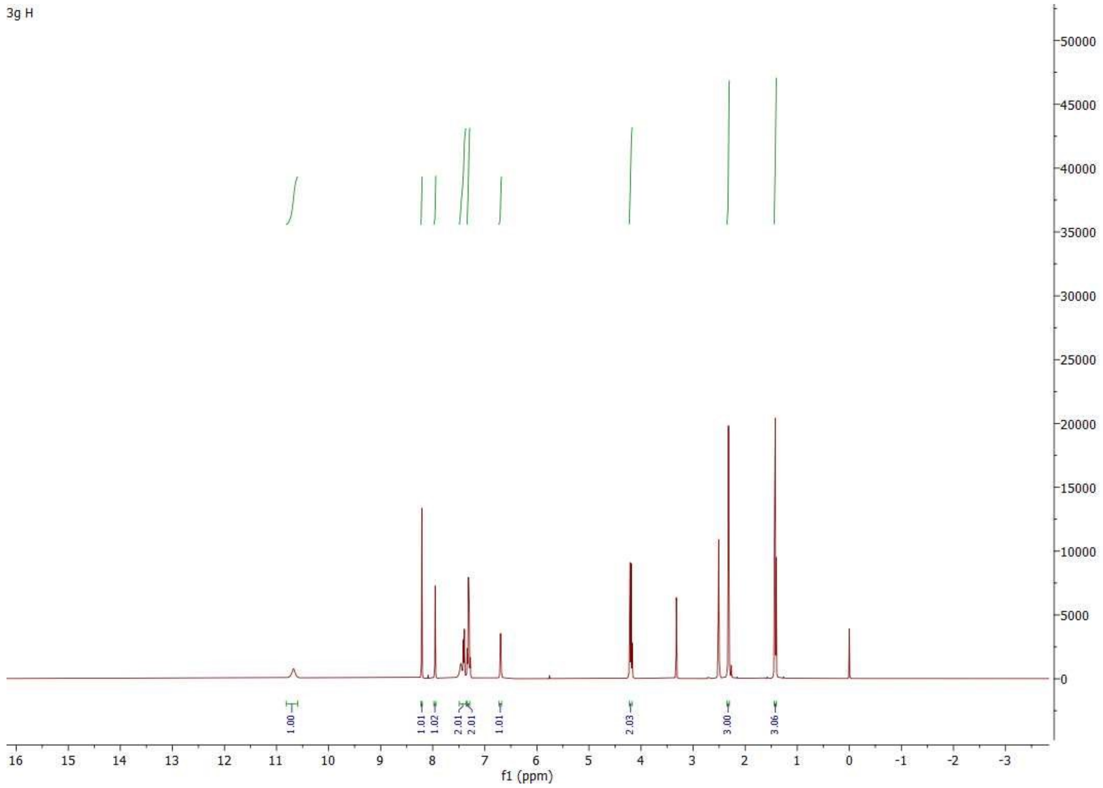

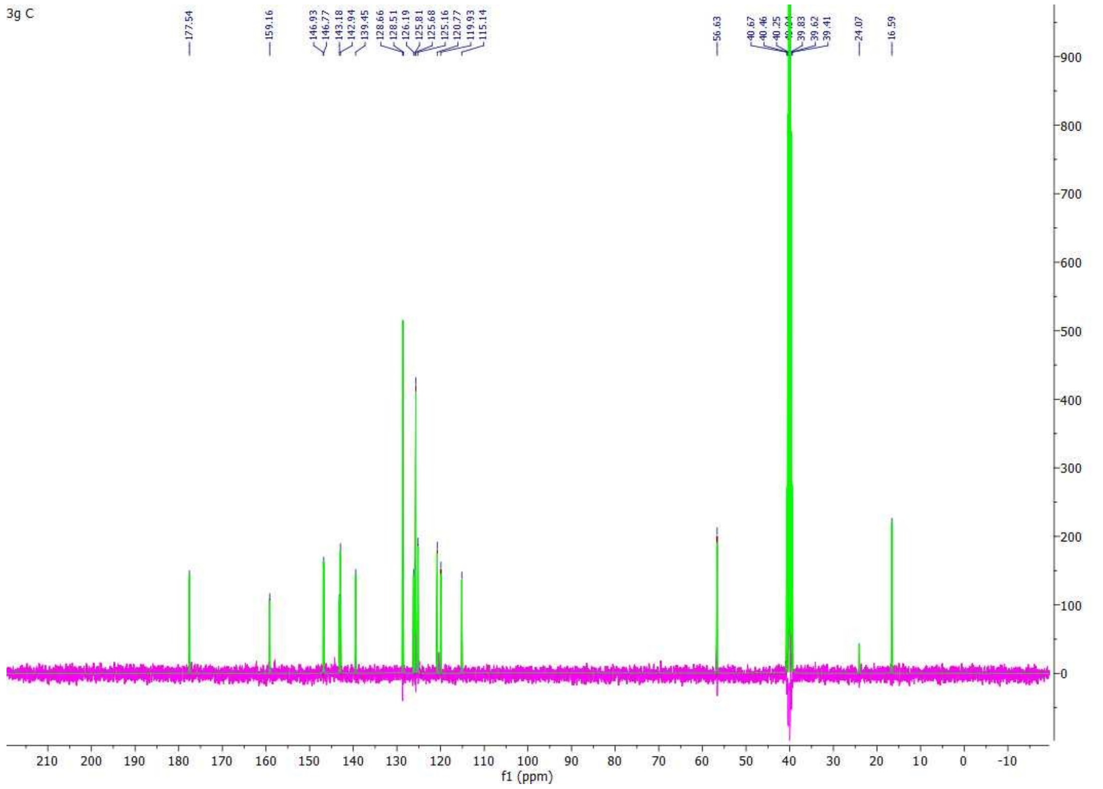

H[1] & ^{13}CNMR Spectrum of Compound 5a

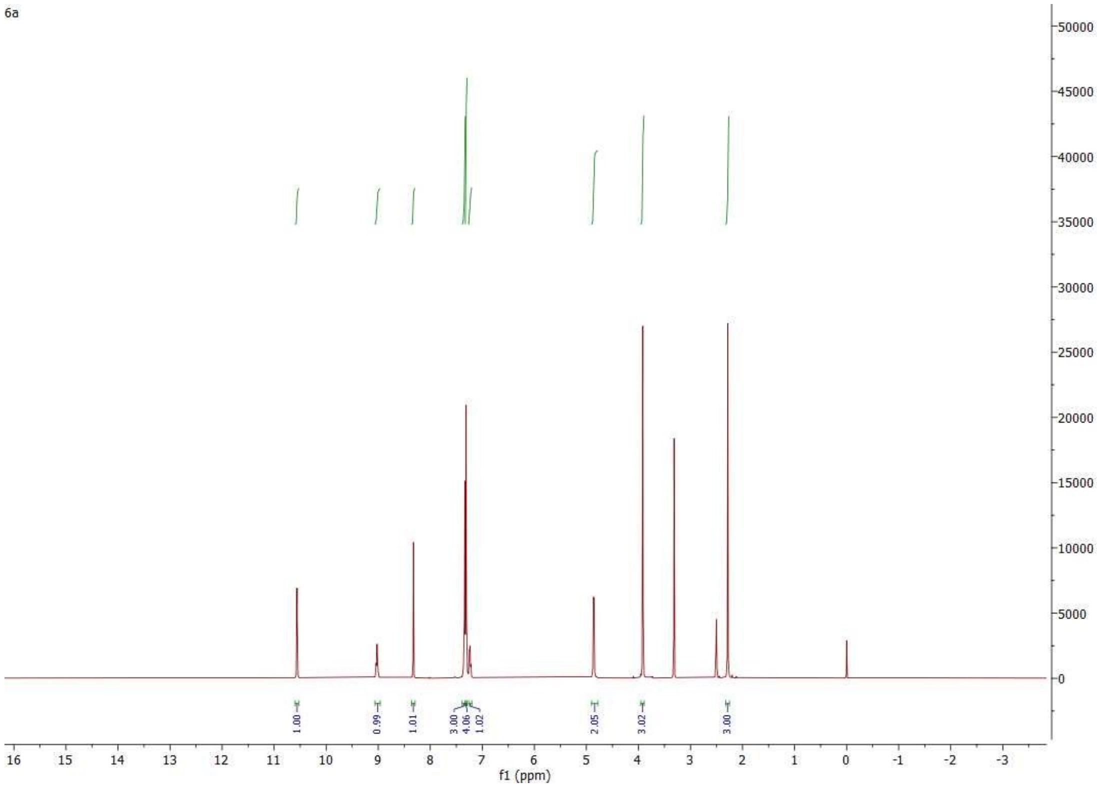

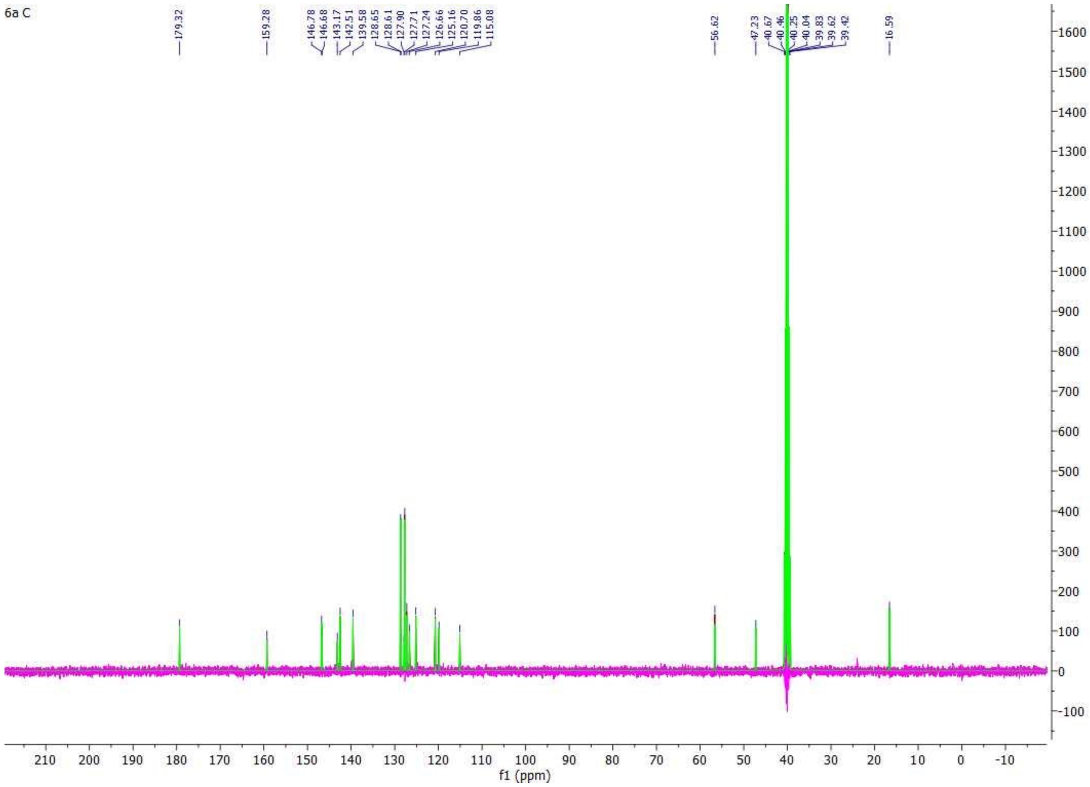

H[1] & ^{13}CNMR Spectrum of Compound 5b

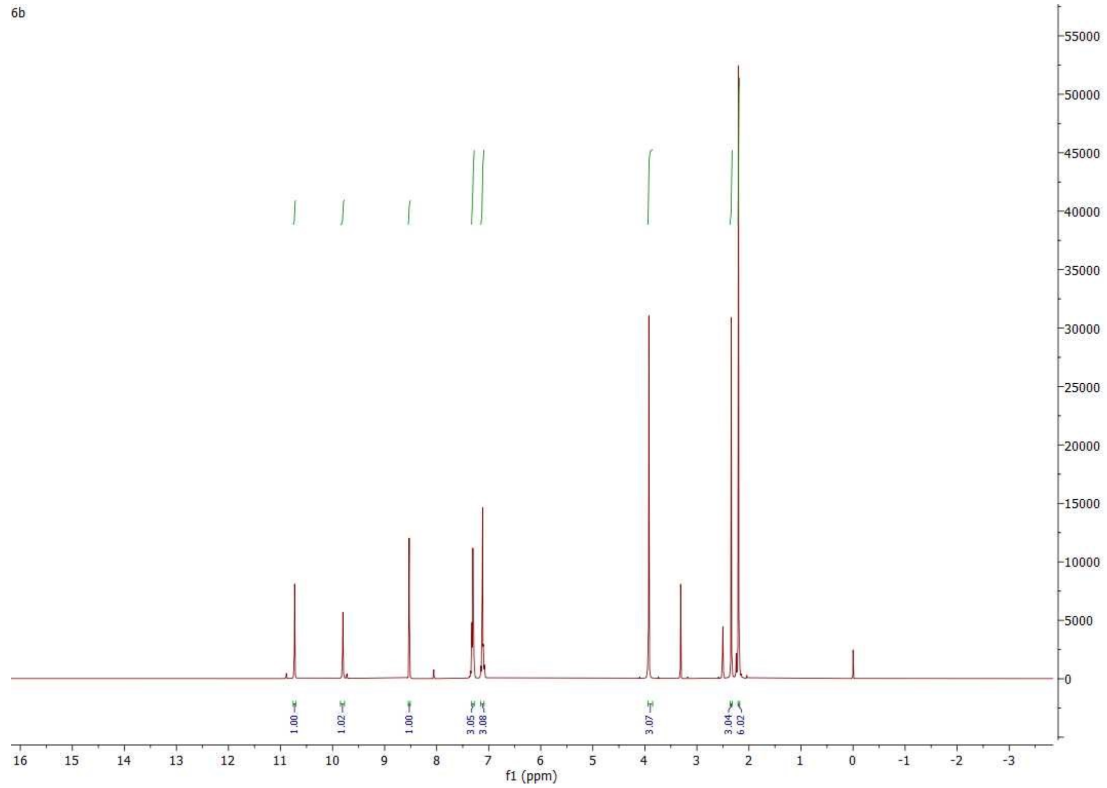

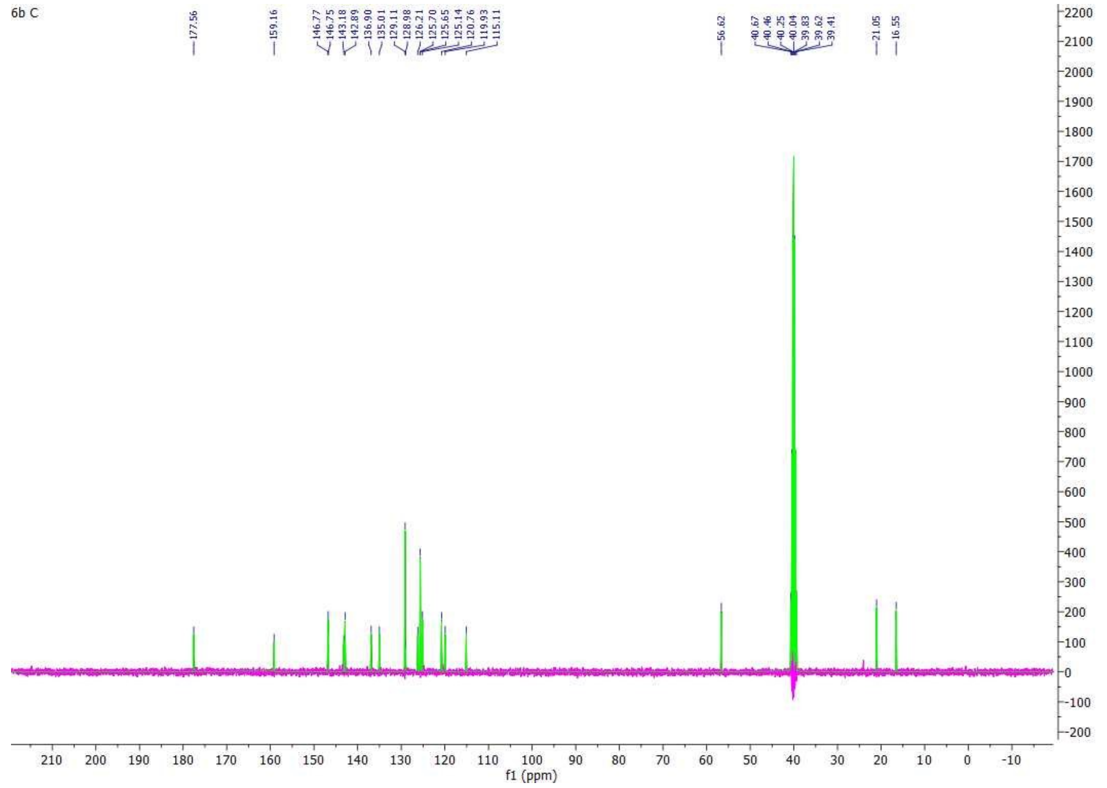

H^1 & ^{13}CNMR Spectrum of Compound 5c

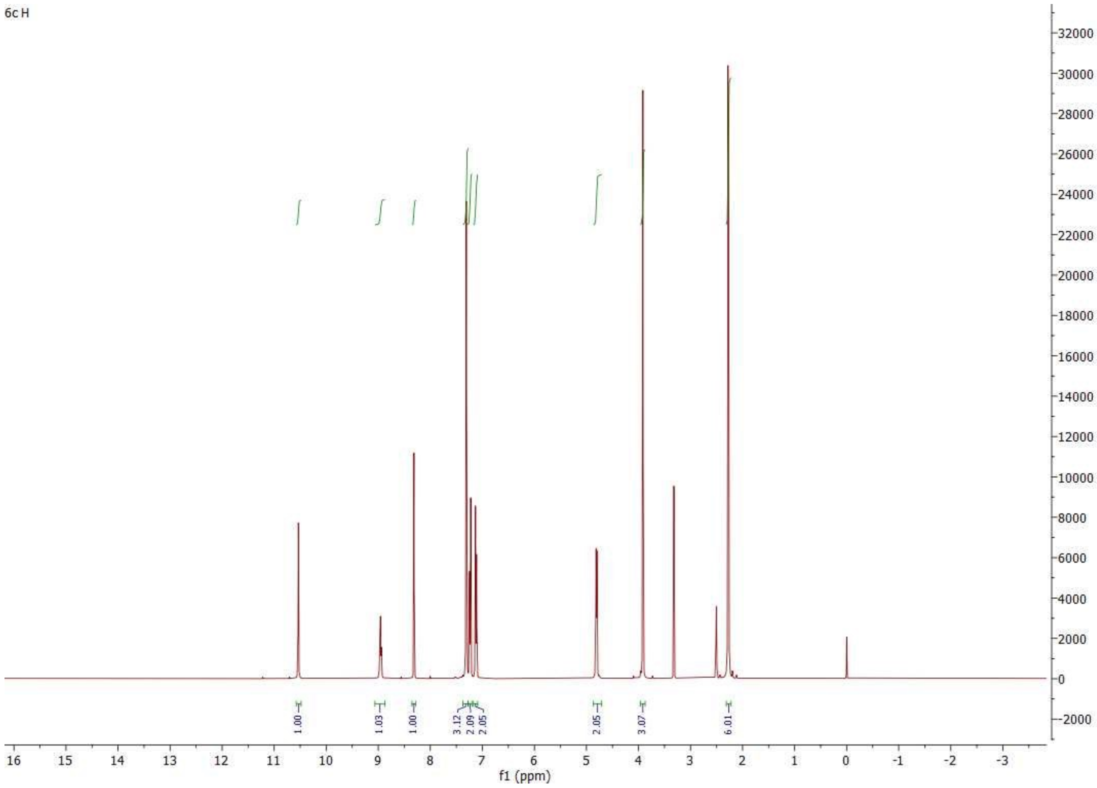

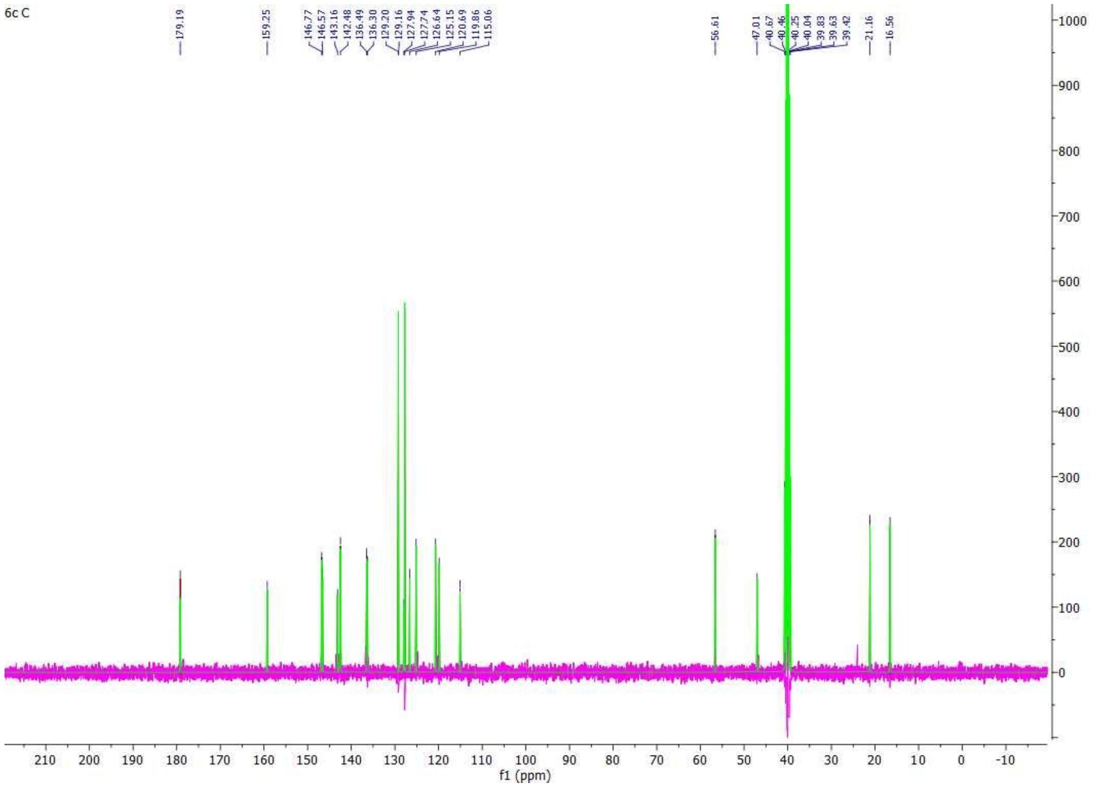

H[1] & ^{13}CNMR Spectrum of Compound 5d

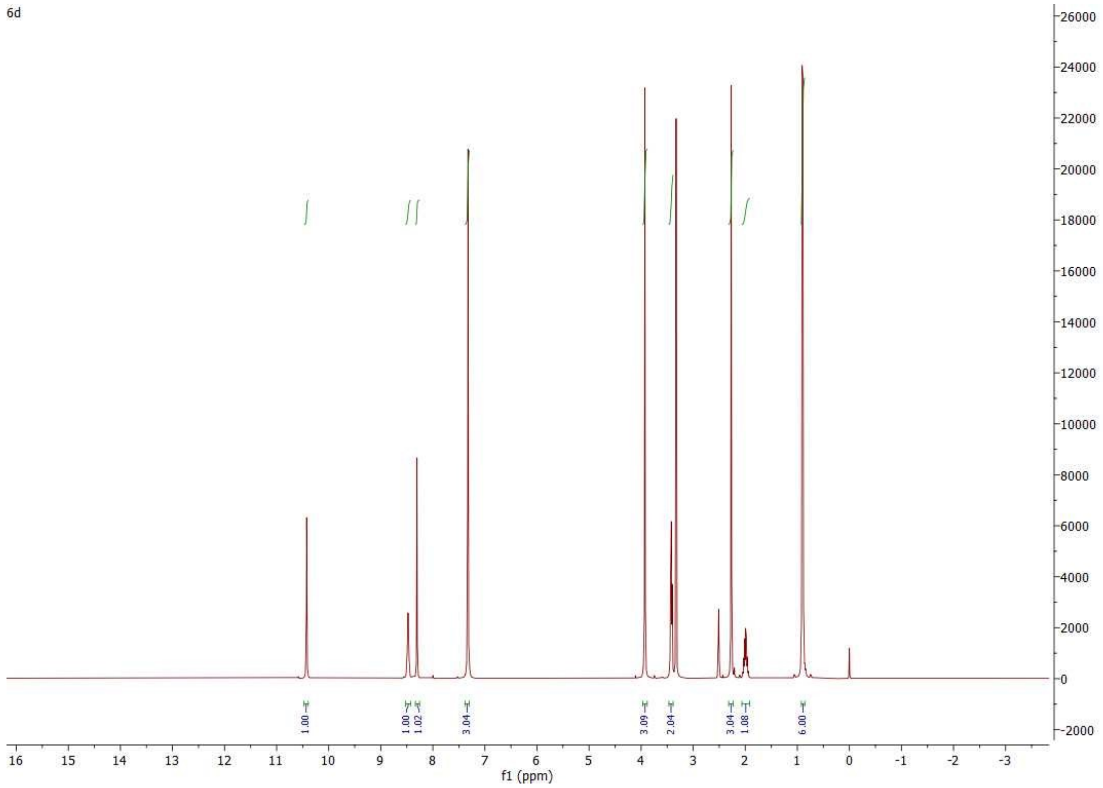

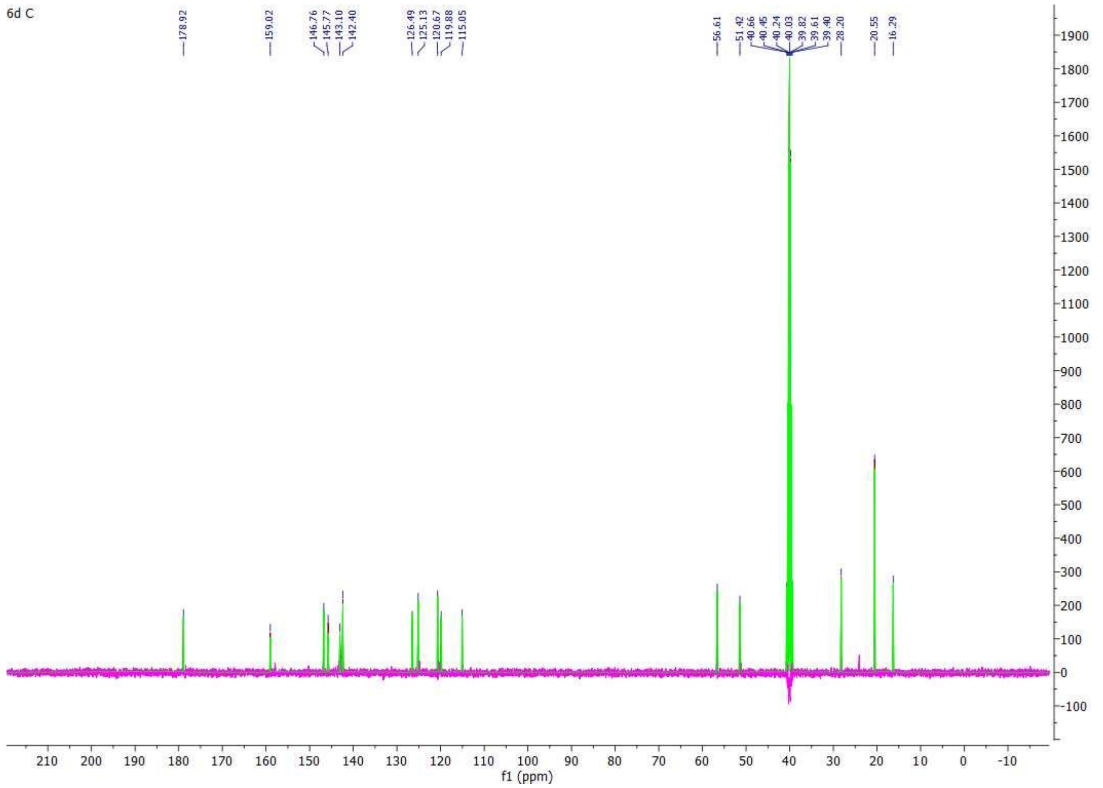

H[1] & ^{13}CNMR Spectrum of Compound 5e

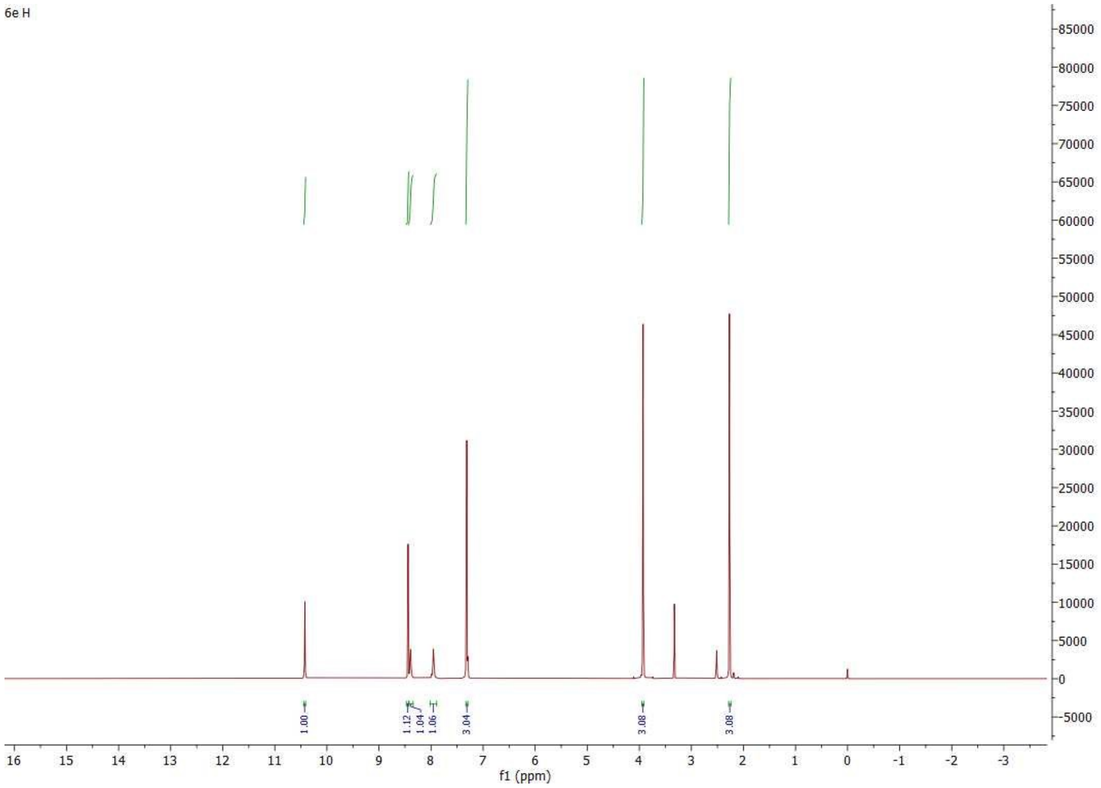

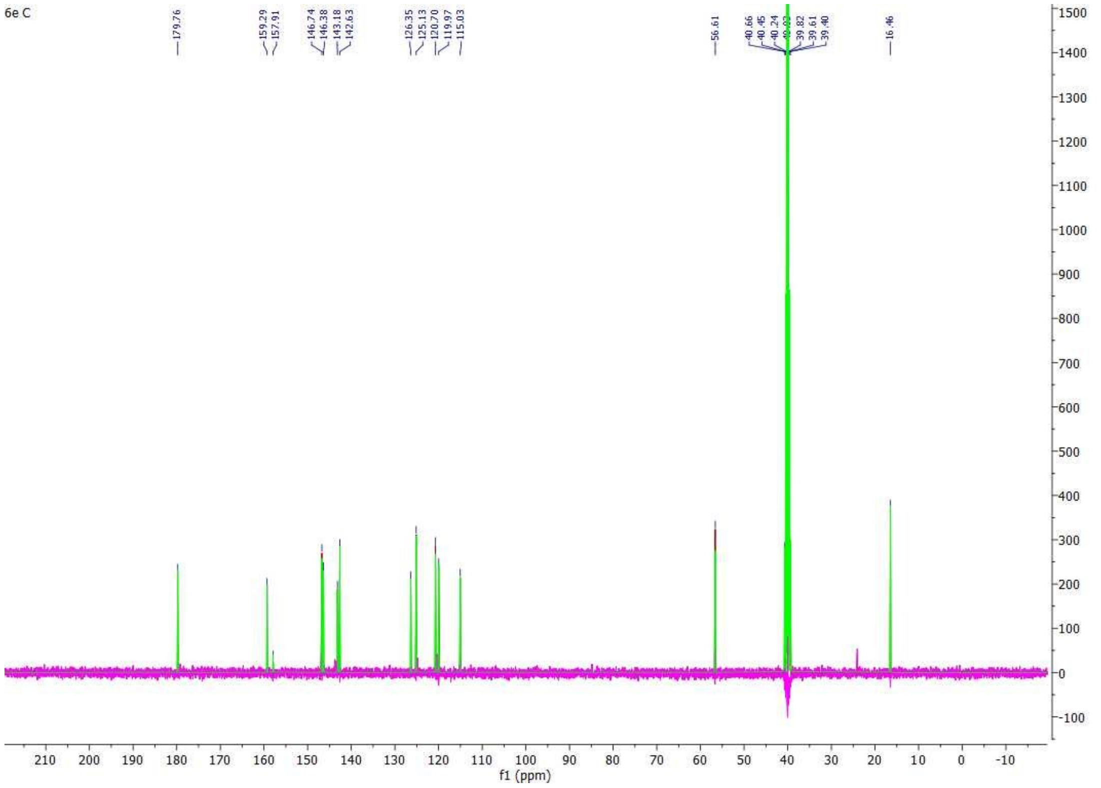

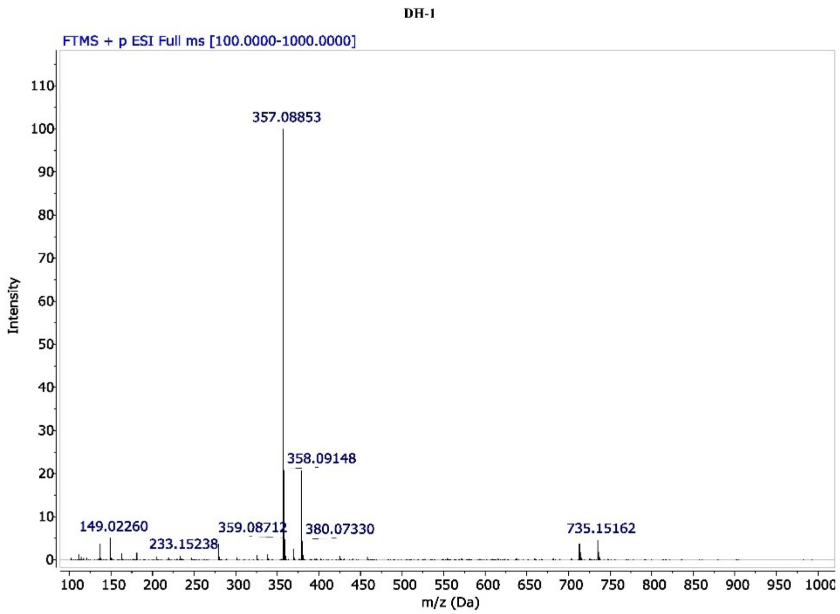

HRMS of compound 3a

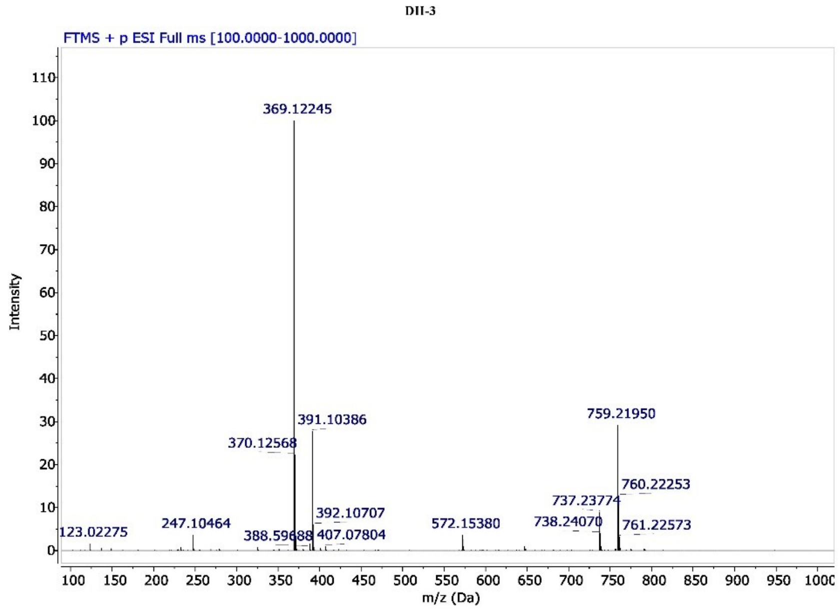

HRMS of compound 3b

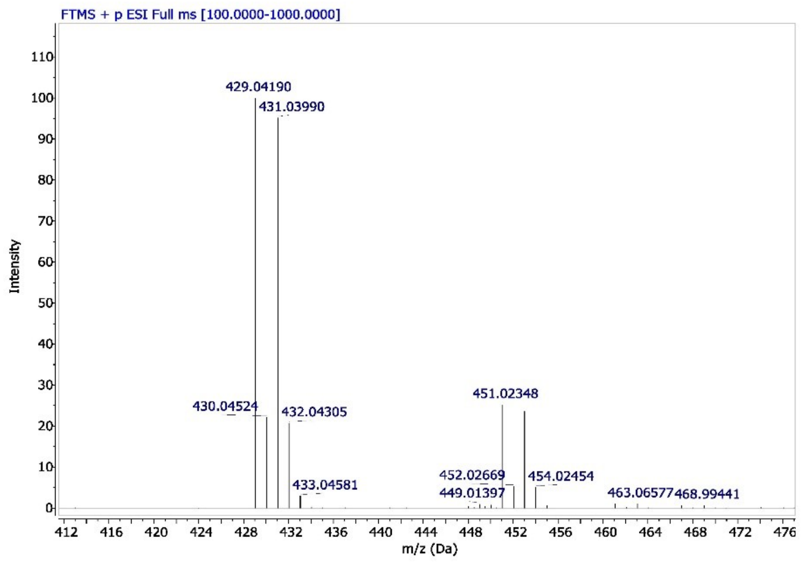

HRMS of compound 3c

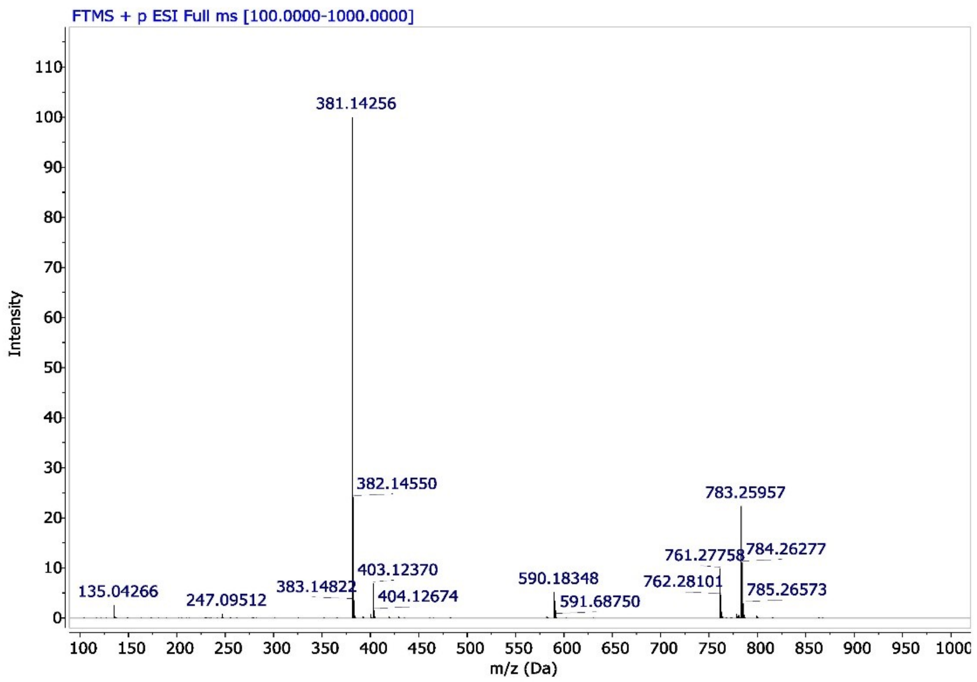

HRMS of compound 3d

DH-5

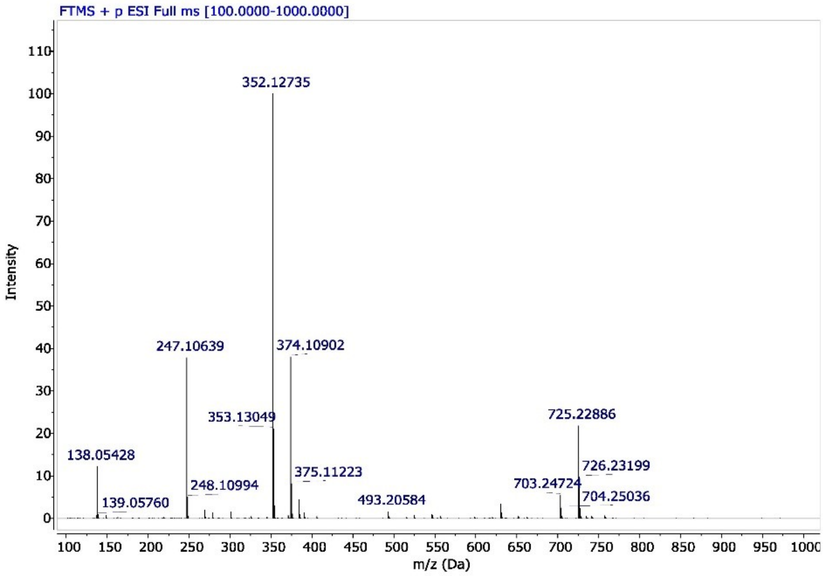

HRMS of compound 3e

DH-9

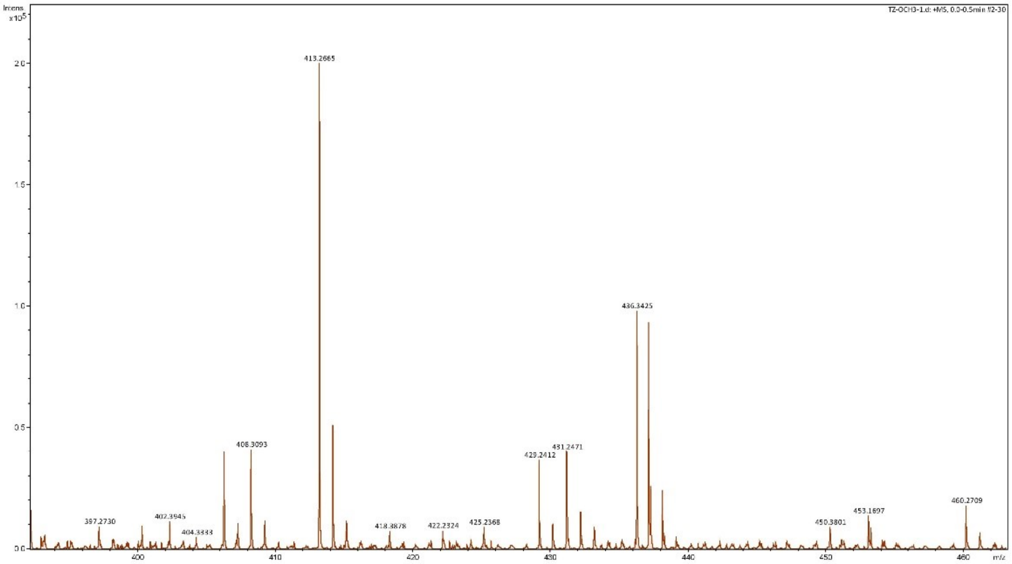

DH-10

HRMS of compound 3f

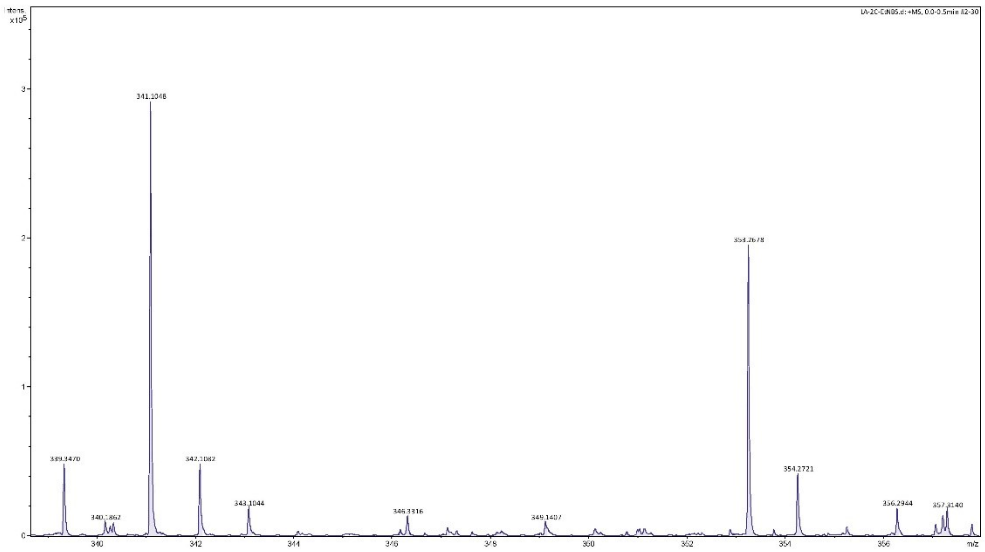

HM-11

HRMS of compound 3g

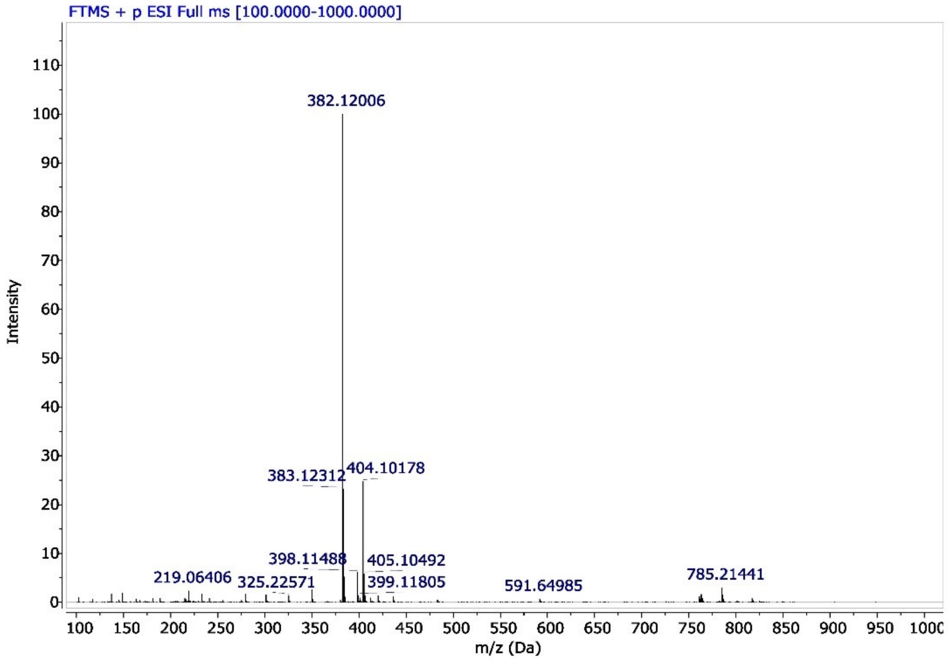

HM-12

HRMS of compound 5a

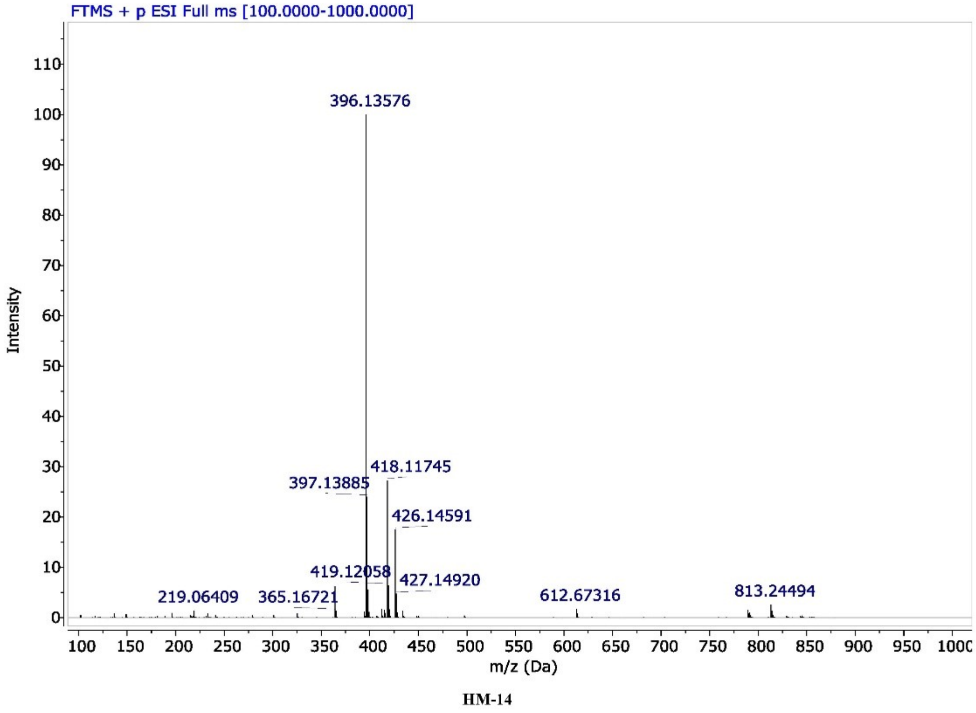

HRMS of compound 5b

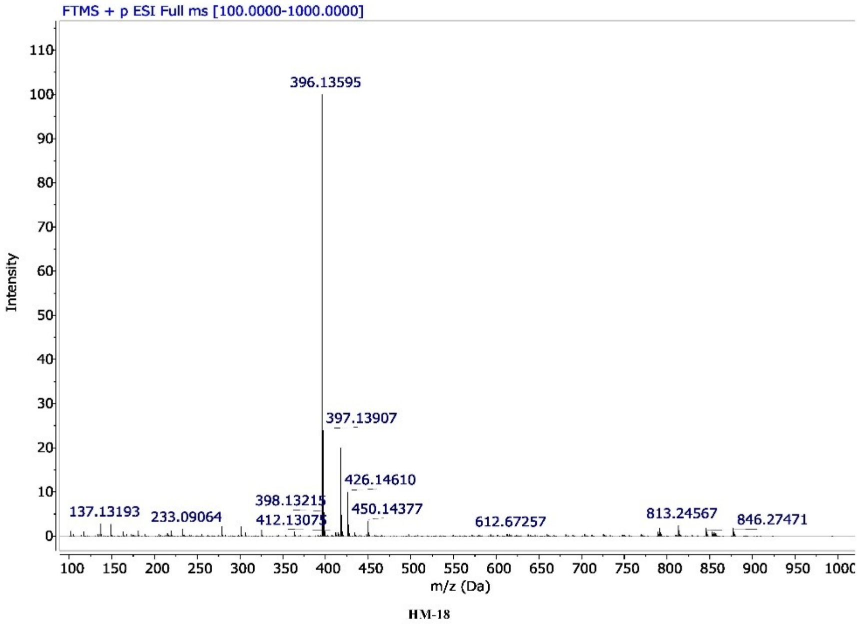

HRMS of compound 5c

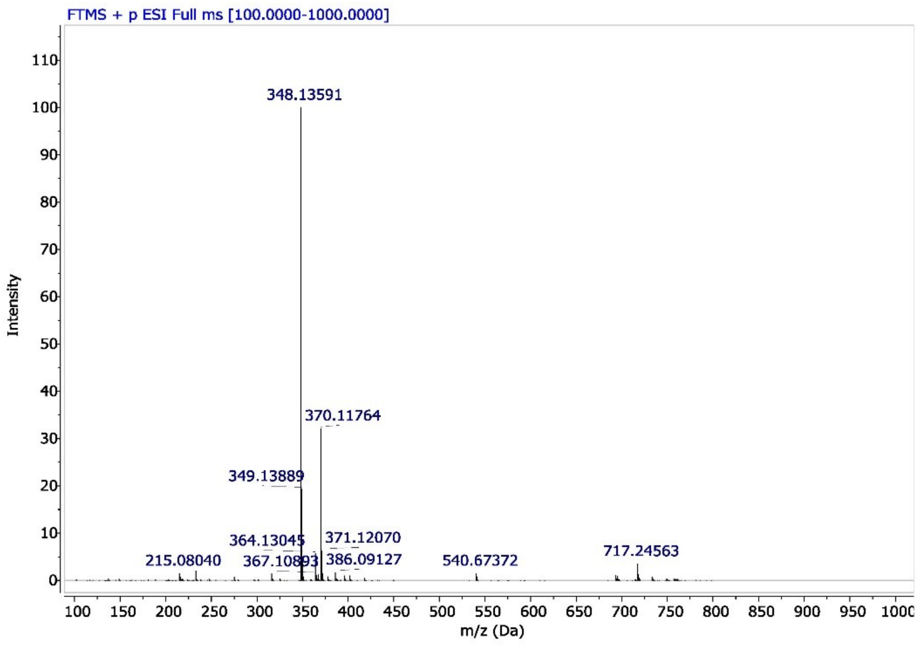

HM-20

HRMS of compound 5d

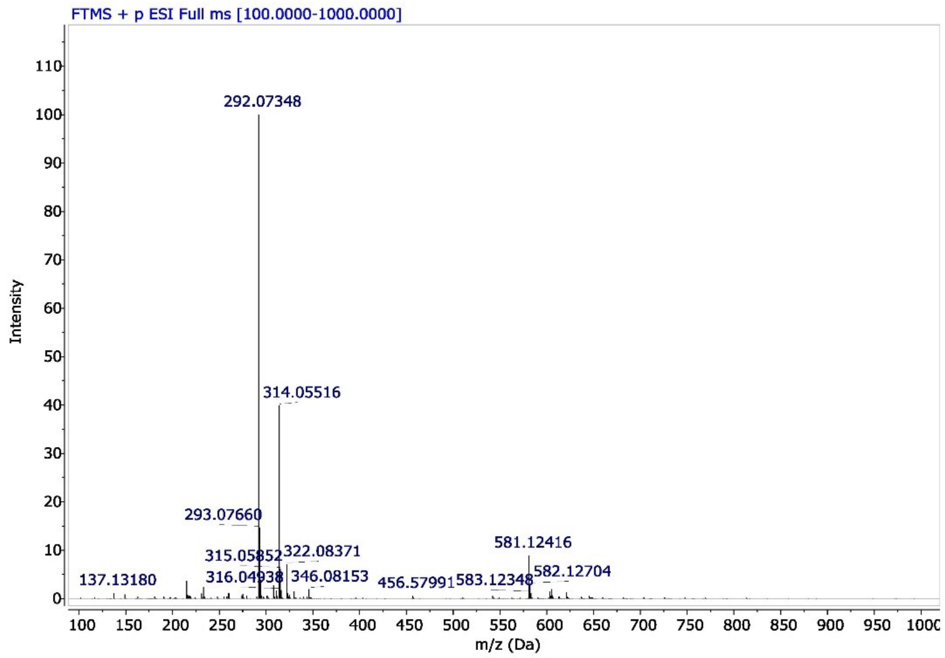

HRMS of compound 5e

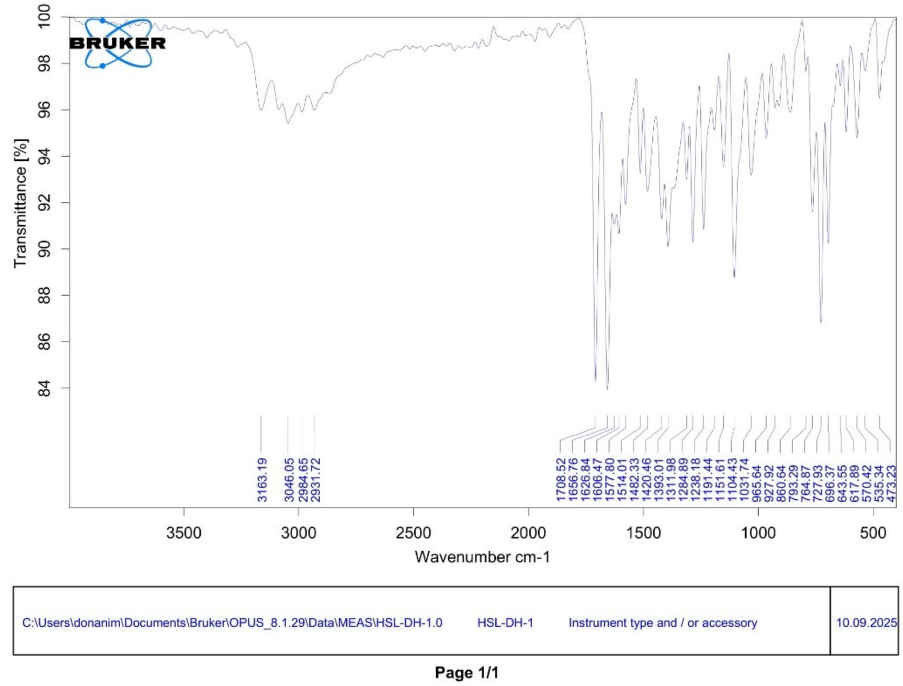

IR spectrum of 3a

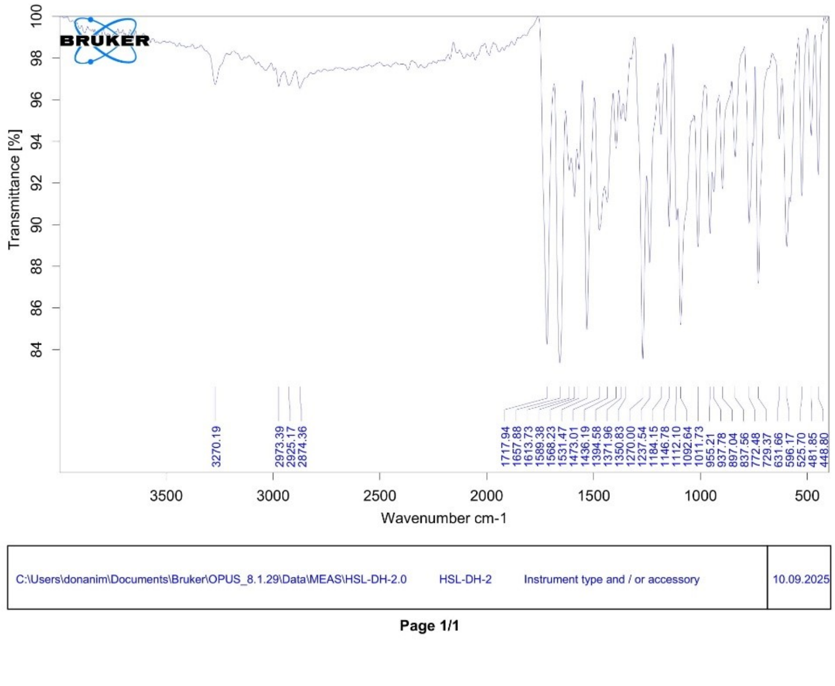

IR spectrum of 3b

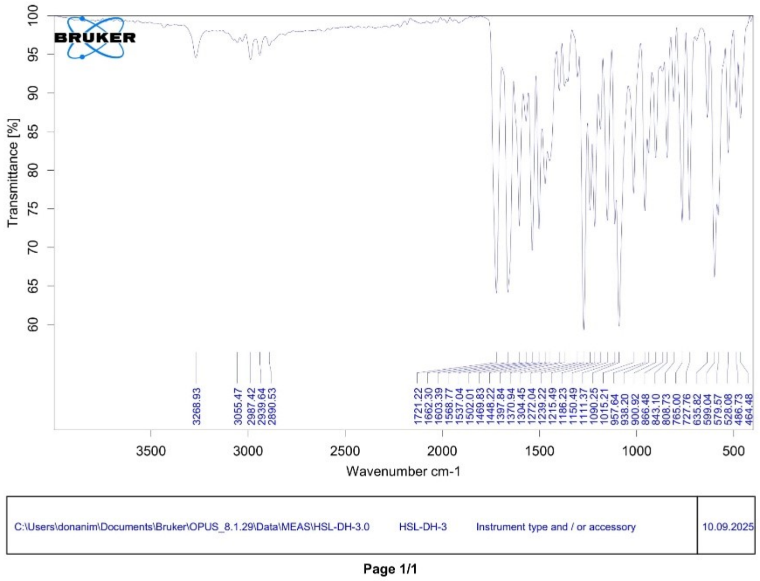

IR spectrum of 3c

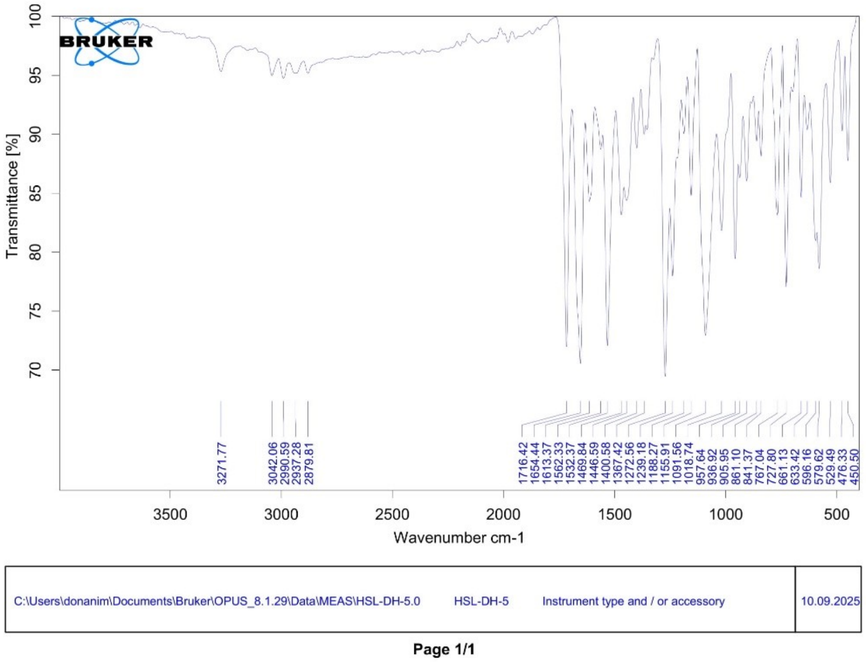

IR spectrum of 3d

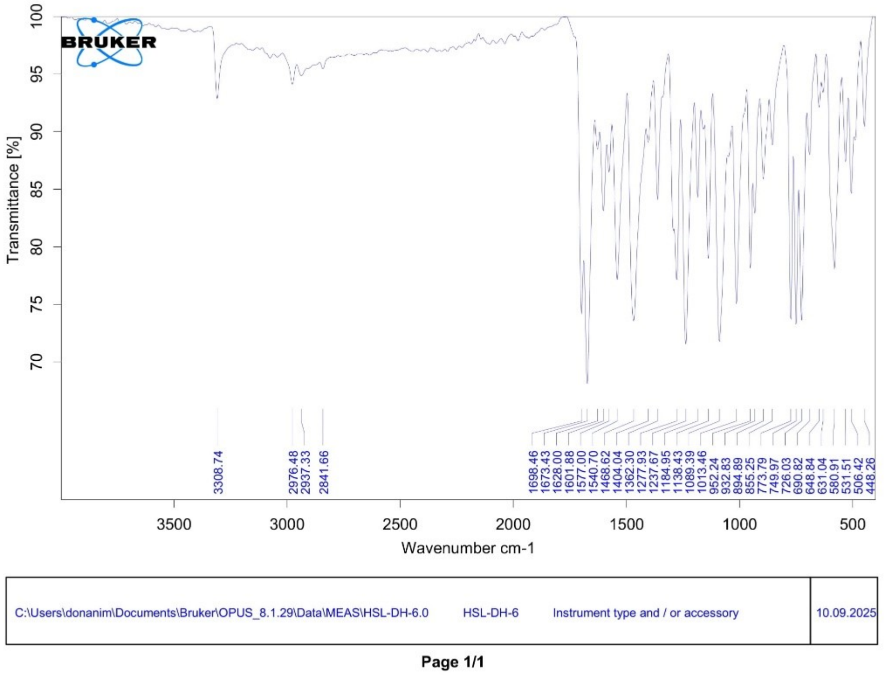

IR spectrum of 3e

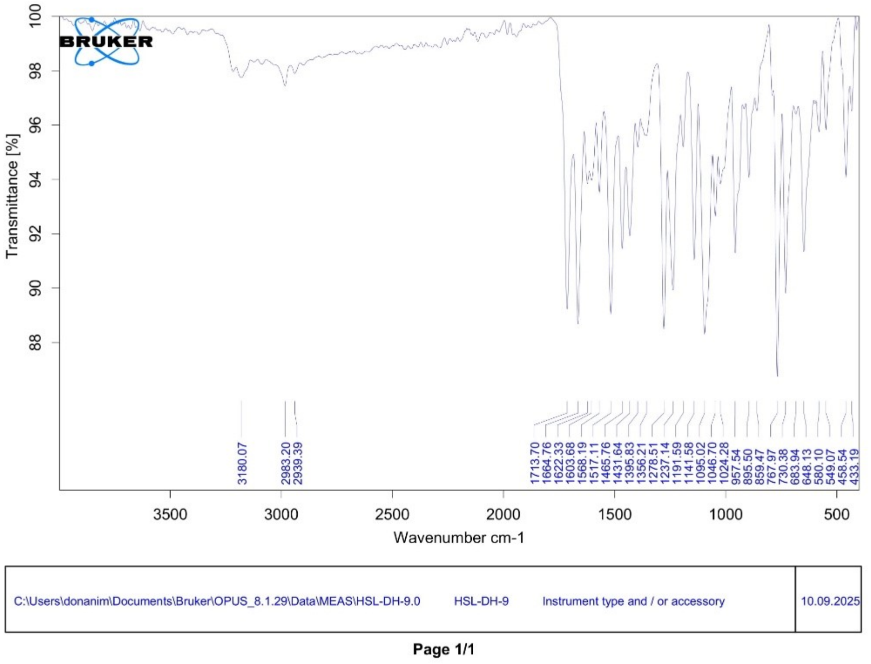

IR spectrum of 3f

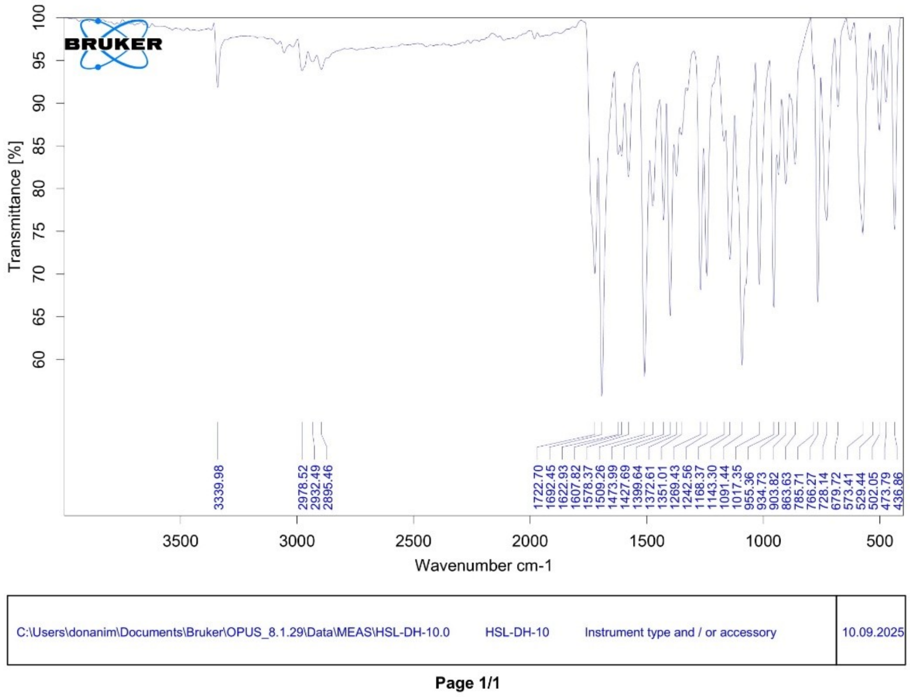

IR spectrum of 3g

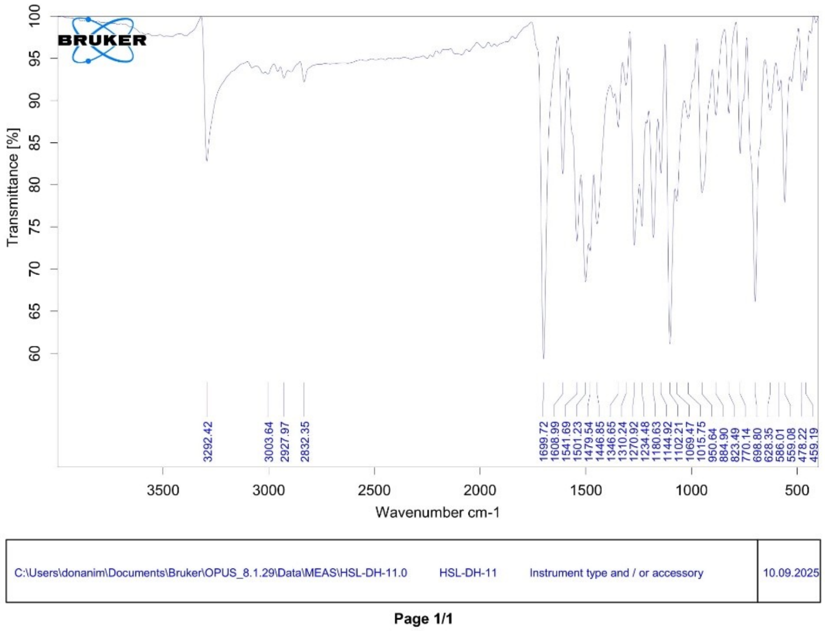

IR spectrum of 5a

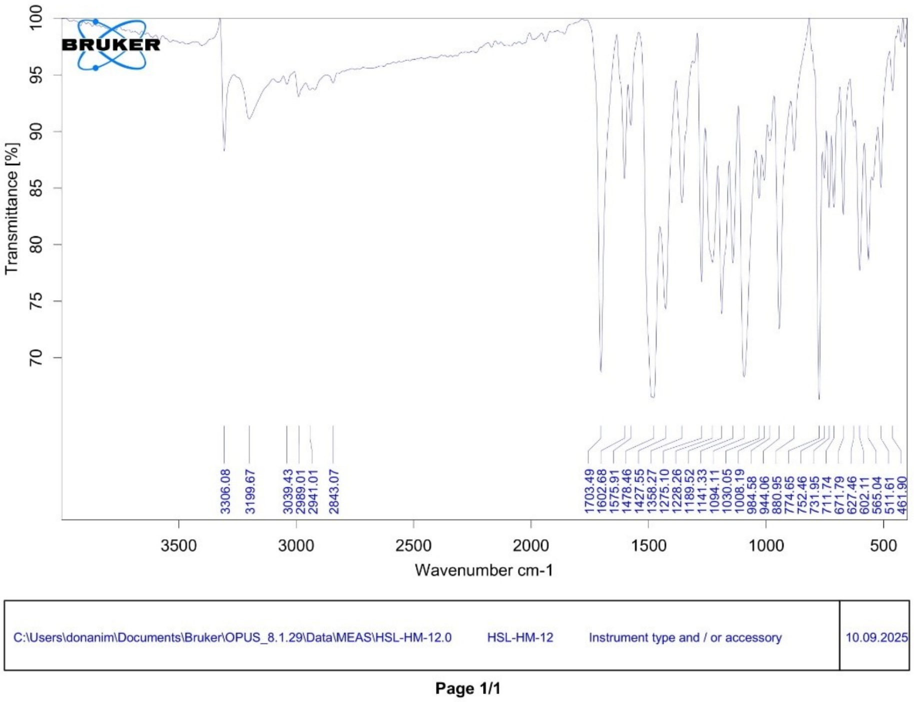

IR spectrum of 5b

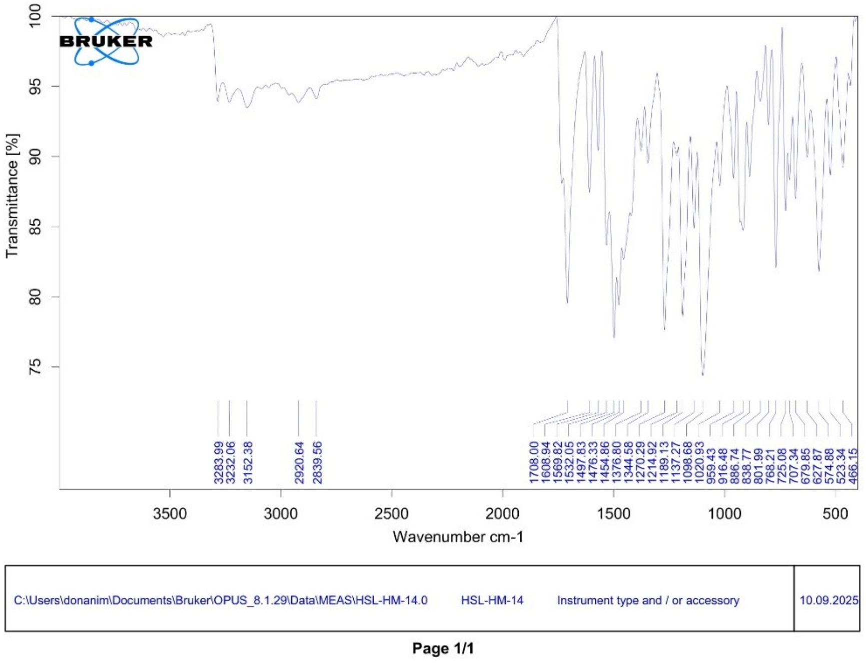

IR spectrum of 5c

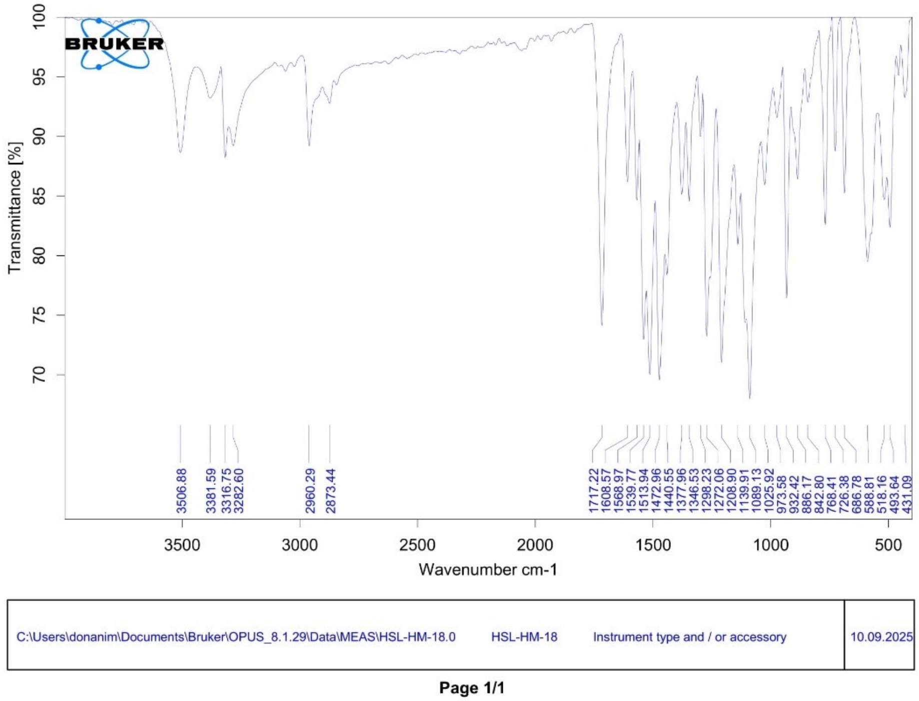

IR spectrum of 5d

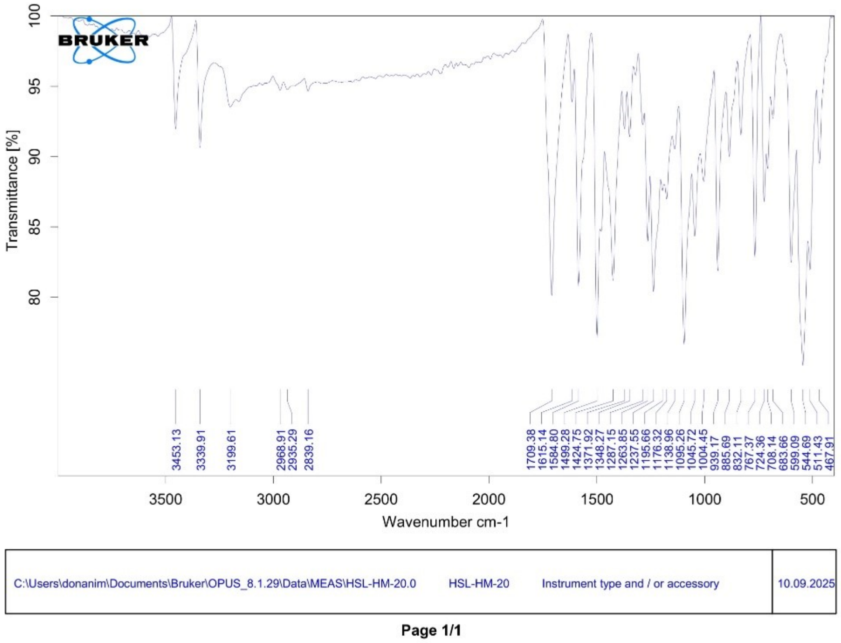

IR spectrum of 5e

UV-vis Stability of Compounds most active compounds 3b and 5e in DMSO

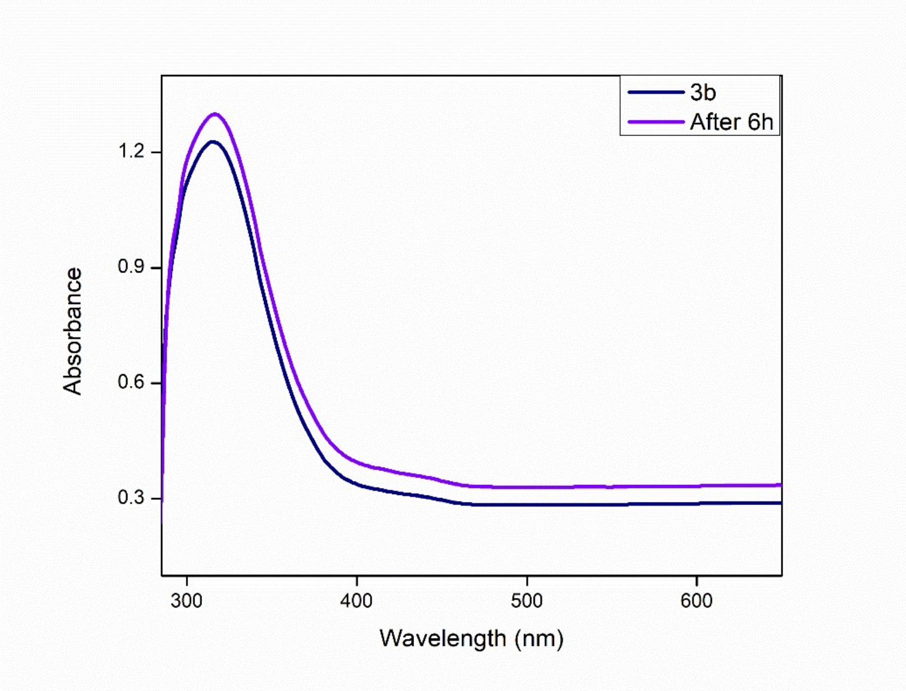

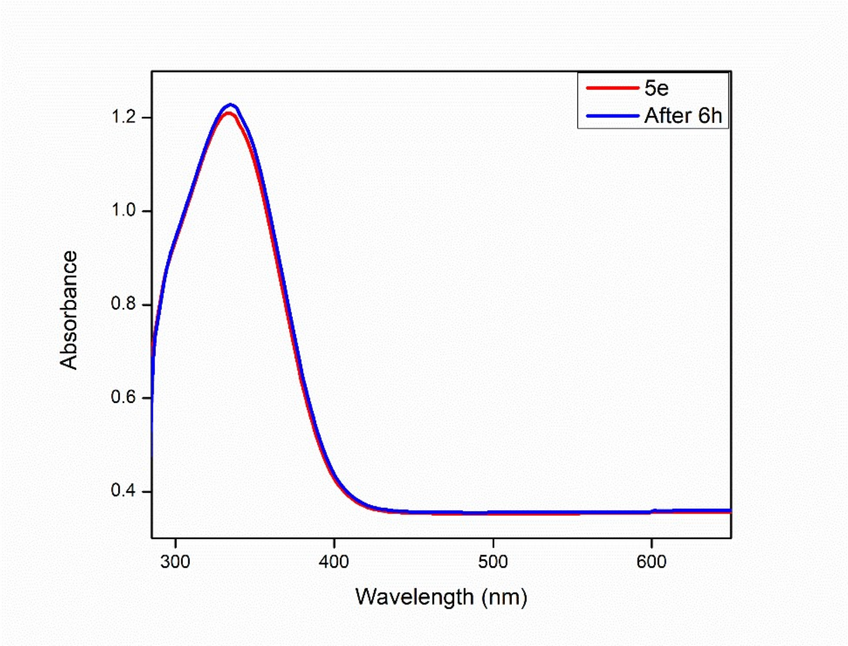

Supplement: RA-015-D5RA04619J-s001 [file RA-015-D5RA04619J-s001.pdf]
